# Metabolic regulation of the maize rhizobiome by benzoxazinoids

**DOI:** 10.1038/s41396-019-0375-2

**Published:** 2019-02-22

**Authors:** T. E. Anne Cotton, Pierre Pétriacq, Duncan D. Cameron, Moaed Al Meselmani, Roland Schwarzenbacher, Stephen A. Rolfe, Jurriaan Ton

**Affiliations:** 1 Department of Animal and Plant Sciences, The University of Sheffield, Sheffield, S10 2TN UK; 2 Department of Animal and Plant Sciences, Plant Production and Protection (P3) Institute for Translational Plant & Soil Biology, The University of Sheffield, Sheffield, S10 2TN UK; 3 Department of Animal and Plant Sciences, biOMICS Facility, The University of Sheffield, Sheffield, S10 2TN UK; 4 Department of Molecular Biology and Biotechnology, The University of Sheffield, Sheffield, S10 2TN UK; 5 Present Address: UMR1332 Biologie du Fruit et Pathologie/Équipe Métabolisme, INRA de Bordeaux & Université de Bordeaux, F-33883 Villenave d’Ornon, France

**Keywords:** Microbiome, Metabolomics

## Abstract

The rhizobiome is an important regulator of plant growth and health. Plants shape their rhizobiome communities through production and release of primary and secondary root metabolites. Benzoxazinoids (BXs) are common tryptophan-derived secondary metabolites in grasses that regulate belowground and aboveground biotic interactions. In addition to their biocidal activity, BXs can regulate plant–biotic interactions as semiochemicals or within-plant defence signals. However, the full extent and mechanisms by which BXs shape the root-associated microbiome has remained largely unexplored. Here, we have taken a global approach to examine the regulatory activity of BXs on the maize root metabolome and associated bacterial and fungal communities. Using untargeted mass spectrometry analysis in combination with prokaryotic and fungal amplicon sequencing, we compared the impacts of three genetic mutations in different steps in the BX pathway. We show that BXs regulate global root metabolism and concurrently influence the rhizobiome in a root type-dependent manner. Correlation analysis between BX-controlled root metabolites and bacterial taxa suggested a dominant role for BX-dependent metabolites, particularly flavonoids, in constraining a range of soil microbial taxa, while stimulating methylophilic bacteria. Our study supports a multilateral model by which BXs control root–microbe interactions via a global regulatory function in root secondary metabolism.

## Introduction

The root-associated microbiome influences plant development and health. These impacts vary from detrimental effects by soil-borne pathogens to beneficial interactions that improve host nutrient acquisition, abiotic stress tolerance and resistance against pests and diseases [1]. A range of biotic and abiotic factors influence the composition and diversity of the rhizobiome [2, 3]. Plant genotype is particularly important [4–10] and it has been proposed that genotypic differences in root chemistry are strongly influential [2, 11]. This is supported by studies showing that mutations affecting root chemistry alter rhizosphere bacterial communities [12–14]. However, the exact genetic and biochemical mechanisms driving these effects remain poorly understood. Elucidating the genetic control of rhizobiome assembly is therefore regarded as an important research goal [15, 16].

**Fig. 1:**
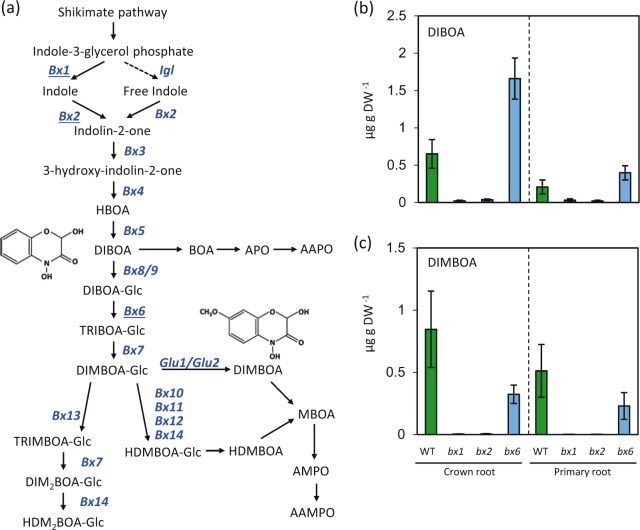
Impact of three independent *Bx* gene mutations on BX production. **a** Benzoxazinoid (BX) production and degradation pathways in maize (*Zea mays*). Names of genes encoding BX biosynthesis enzymes are shown in blue. Genes investigated in this study are marked by underlined letter fonts. **b** Concentrations of DIBOA and DIMBOA in different maize genotypes and tissue types. Data represent means (±SE; *n* = 6)

Benzoxazinoids (BXs) are emerging as major regulatory compounds of biotic interactions [17–20]. BXs are tryptophan-derived heteroaromatic metabolites with benzoic acid moieties that are produced in large quantities by roots of the *Poaceae*, including the cereal crops maize, wheat and rye [21]. The concentration of BXs can differ between root types of the same plant. In maize, total BX concentrations are higher in crown roots (originating from the stem) than primary roots (developing from the radicle) [20, 22]. Previous studies have shown that BXs and their breakdown products are biocidal to some soil-borne bacteria and fungi, but act as recruitment signals for others, such as the plant growth-promoting rhizobacterial strain *Pseudomonas putida* KT2440 [19, 23]. Hence, BXs act as important regulators of below-ground plant–microbe interactions, which can vary between different root types. Although the metabolic pathways involved in the production and degradation of BXs are well characterised (Fig. 1a), most studies on the effects of BXs on plant–biotic interactions have focussed on individual organisms, and rarely considered different root tissues. Therefore, the wider impact of BXs on complex rhizobiome communities has remained unknown [24].

A recent study by Hu et al. [25] reported that the microbial community structure of soil from BX-producing wild-type (WT) maize differs from that of a BX-deficient *bx1* mutant of maize [25]. These BX-dependent changes in soil microbiome were associated with resistance-inducing activity in plants cultivated on soil from BX-producing WT plants. In addition, Hu et al. [25] identified the BX catabolite 6-methoxy-benzoxazolin-2-one (MBOA) as a root-derived compound from BX-producing WT maize that remains stable in soil for several months, and which can complement soil from the *bx1* mutant for resistance-inducing activity. Accordingly, it was concluded that accumulation of MBOA was responsible for the selection of a resistance-inducing soil microbiome. However, the results by Hu et al. [25] can also be explained by a mechanism whereby BXs and associated derivates (e.g. MBOA) act as external  signalling molecules that stimulate the production of a wider range of soil-conditioning root metabolites. An equivalent regulatory role has been reported for the BX precursor indole during aboveground tri-trophic interactions of maize, where the release of this volatile stimulates systemic emission of a wider range of herbivore-induced volatiles, which in turn recruit plant-protecting parasitoids [26].

Support for a signalling role of BX metabolites in maize–biotic interactions comes from reports that the BX metabolite 2,4-dihydroxy-7-methoxy-1,4-benzoxazin-3-one (DIMBOA) can act as an apoplastic defence signal controlling cell wall defences against fungi and aphids [17, 18]. It is thus plausible that the BX pathway has an additional impact on below-ground microbial communities via the regulation of defence-related root metabolism. To date, few studies have explored the impacts of mutations in single plant genes on root-associated microbial communities, and none of these have characterised the associated changes in metabolic root profiles, making elucidation of the underpinning mechanisms challenging [14, 24, 25, 27]. Addressing this challenge requires an integrated and global approach that combines metataxonomic profiling with untargeted metabolomic analysis.

Considering the variable quantities of BXs in different root types [20] and their regulatory role in plant defence [17, 18], we hypothesise that the impacts of BXs on the rhizobiome differ between root types, and are partially driven by their activity as  regulatory signals of plant  secondary metabolism. To address these hypotheses, we have analysed the effects of *bx* mutations on root metabolism and root-associated bacterial and fungal rhizobiome communities of maize. Using untargeted mass spectrometry analysis in combination with rRNA gene and internal transcribed spacer (ITS) sequencing, we have compared the effects of three mutations in different steps of the BX biosynthesis pathway (*bx1*, *bx2* and *bx6*; Fig. 1a) to establish relationships between *Bx*-regulated root metabolites and *Bx*-dependent rhizosphere microbiota.

## Methods

### Plants, growth substrate and growth conditions

Experiments were conducted with WT maize (*Zea mays* cv. W22) and *Ds* transposon insertion lines of W22 in *Bx1*, *Bx2* and *Bx6* (Fig. S1), as described previously [28]. Sterilised seeds were pre-germinated and planted (1 seed/750 ml pot) in a 3:1 (v:v) mixture of agricultural soil:autoclaved perlite. Details of the soil and plant growth conditions are presented in the Supplementary methods. Since many *Bx* genes are lowly expressed in older plants [29] and exudation of the BX compound DIMBOA declines in maize roots between 1 and 3 weeks after planting [19], root material was collected from 17-day-old plants, thereby guaranteeing BX exudation, while also ensuring sufficient root material for root microbiome and metabolome analysis. Genotypes of WT and mutant plants were verified by PCR of DNA extracts from material collected at the time of harvest (Fig. S1, Table S1a). Further details of plant growth and genotyping methodology can be found in the Supplementary methods.

### Untargeted metabolite profiling of root metabolites

Crown and primary roots were carefully removed from the growth substrate and washed in distilled water before lyophilisation. Metabolite extracts from crown and primary roots were analysed by untargeted ultra performance liquid chromatography quadrupole time of flight mass spectrometry (UPLC-Q-TOF), as described in the Supplementary methods.

### Microbial community profiling

Plants were removed carefully from the growth substrate and placed into sterile Petri dishes. Roots were shaken to remove all but tightly adhering rhizosphere soil, crown and primary roots were separated and then placed in sterile tubes and flash-frozen in liquid nitrogen. For each genotype/tissue combination, root samples from eight independent plants were sampled. Soil samples were obtained from unplanted pots using a 12 mm diameter core. Illumina MiSeq amplicon sequencing, targeting 16S rRNA genes and the ITS2 sequences and surrounding regions, was used to describe the bacterial and fungal microbial communities, respectively, from root and soil samples. Quantitative PCR (qPCR) of DNA extracts was used to compare total bacterial and fungal DNA between WT and mutant plants. For details about DNA extraction, sequencing library preparation, qPCR and data analysis, see the Supplementary methods.

### Correlation analysis of operational taxonomic units and metabolite ions

To identify candidate metabolites that could influence the bacterial communities associated with the roots, we determined correlations between the metabolic profiles (six independent replicates) and microbiome data (eight independent replicates) (Fig. S2). Since metabolome and microbiome analyses are destructive, it was not possible to compare the same samples directly. Accordingly, datasets were paired at random 100 times and the average Spearman correlation was calculated between the variance stabilised values obtained from the DESeq2 generalised linear models. To reduce the number of calculations, only bacterial taxa and metabolites showing statistically significant differences between the WT and any mutant line were considered. Fungal Operational Taxonomic Units (OTUs) were not included in the analysis as few fungi were affected by the *bx* mutations. To further increase confidence levels, only correlations of magnitude >0.5 (±) were selected. Putative identities were assigned to metabolites based on exact mass measurements, using the METLIN and PubChem databases, as reported previously [30, 31]. The scripts used for analysis are available from the authors upon request.

## Results

### Effects of *Bx* genotype on plant growth phenotype and root BX profiles

To exclude indirect developmental effects of the *bx* mutations on root metabolites and associated microbiota, growth phenotype and dry weight of WT and *bx* mutant plant shoots and different root types were compared. All lines were morphologically similar, developed similar biomass and had similar root distributions at the time of analysis (Fig. S3), indicating that the *bx* mutations do not significantly affect growth and development. Quantification of root BXs focused on the aglycones of 2,4-dihydroxy-1,4-benzoxazin-3-one (DIBOA) and DIMBOA, since these are more stable and reliable for quantification than the corresponding glycosides. The concentrations of both DIBOA and DIMBOA were dramatically reduced in both crown and primary roots of the *bx1* and *bx2* mutants compared to WT (Fig. 1b and c). This is consistent with the enzymatic function of *Bx1* and *Bx2* in the first two dedicated steps of the BX pathway (Fig. 1a [32]). Compared to WT plants, roots of *bx6* mutants had increased concentrations of DIBOA and reduced concentrations of DIMBOA (Fig. 1b and c), which is consistent with the DIBOA-glycoside dioxygenase activity of BX6 in the multi-step conversion of DIBOA into DIMBOA (Fig. 1a [33]). However, as previously reported for shoot BX levels in this mutant [28], the impact of the *bx6* mutation was partial: primary and crown roots of *bx6* showed only 62% and 55% reductions in DIMBOA concentrations compared to the WT, respectively. Thus, the impact of the *bx6* mutation on BX biosynthesis was relatively weak and only partially blocked DIMBOA production without majorly affecting total BX concentrations.

### Global impacts of *Bx* genotype on the root metabolome

**Fig. 2:**
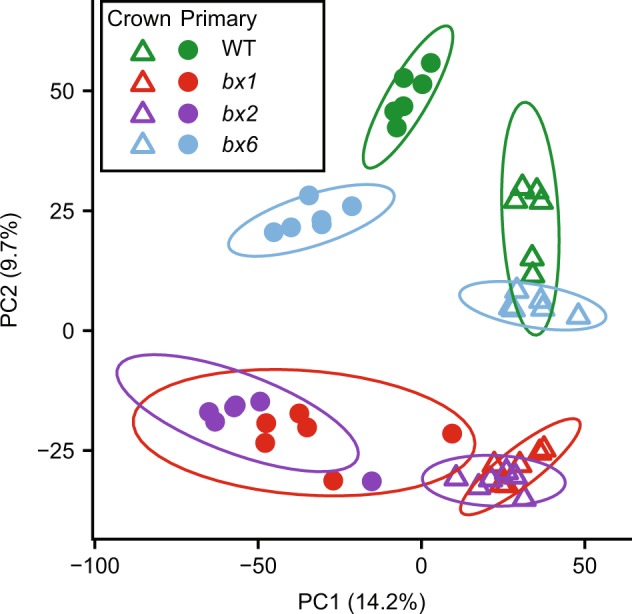
Principal component analysis (PCA) of metabolite ions (positive and negative) extracted from crown and primary roots of wild-type (WT) and *bx* mutants. Ellipses show confidence intervals (*n* = 6)

BXs can influence plant–biotic interactions indirectly through their activity as within-plant defence signalling compounds [17, 18]. Accordingly, it is possible that mutations in *Bx1*, *Bx2* and *Bx6* affect a wider set of root metabolites than BXs which, in turn, could influence the composition of the rhizobiome. To examine the impacts of the three *bx* mutations on the wider root metabolome, we profiled methanol extracts from crown and primary roots by UPLC-Q-TOF mass spectrometry. This untargeted analysis identified a total of 22,868 ions between all tissue/genotype combinations (6411 ESI− and 16,457 ESI+). Unsupervised PCA of all ions revealed that both root type and genotype had major impacts on the metabolite profiles of maize roots (Fig. 2; Table S2). Component 1, explaining 14.2% of the variation in the data, predominantly separated samples from crown and primary roots, which was more pronounced for WT and *bx6* plants than for *bx1* and *bx2* plants. Component 2, which explained 9.7% of the variation, separated WT and *bx6* samples from *bx1* and *bx2* samples, which is consistent with our finding that the *bx1* and *bx2* mutations have similar impacts on total BX production, whereas the *bx6* mutation has a relatively minor effect on root BX composition (Fig. 1b and c). Statistical analysis of the metabolite samples confirmed a significant effect of root type, genotype, and root type × genotype interaction, which together explained 43% of the variation in the data (Table S2). A generalised linear model, assuming a log-normal distribution of ion abundance, was used to identify metabolites that differed between samples. Fig. S4 presents all differentially abundant ions between WT and *bx* mutant roots (crown and primary roots) and the overlap between these sets. Together, the untargeted metabolic profiling of WT and *bx* roots shows that the *bx1* and *bx2* mutations have major impacts on the root metabolome, indicating a global function of BXs in metabolic regulation and differentiation of maize roots.

### Global effects of roots and *Bx* genotype on soil bacterial and fungal communities

To examine impacts of maize roots, root type and *Bx* genotype on microbial communities, DNA was extracted from rhizosphere soil-covered crown and primary roots and plant-free soil. Quantification of total bacterial 16S rRNA genes and fungal ITS regions by qPCR revealed no statistically significant differences between WT and mutant plant samples (Fig. S5), indicating that *Bx* genotype and root tissue did not affect total microbial biomass. To investigate whether root type and *Bx* genotype changed microbiome composition, bacterial 16S rRNA genes and fungal ITS2 regions were amplified and subjected to Illumina sequencing. Following quality control and chimera removal, the bacterial dataset consisted of 8,740,090 16S rRNA gene sequences, with a mean sequence length after primer removal of 377 bp and 52,176–234,324 sequences per sample (Table S3). The fungal dataset consisted of 9,722,174 ITS sequences with trimmed sequence lengths of 130 bp after primer removal and 45,382–280,805 sequences per sample (Table S3). Sequences were classified into 41,449 bacterial OTUs and 21,981 fungal OTUs. Rarefaction analysis indicated sufficient sequencing depth to capture the majority of OTUs for both bacteria and fungi in all samples (Fig. S6). Dominant bacterial phyla included the proteobacteria (43.4%) and actinobacteria (29.0%). At the class level, bacterial OTUs included betaproteobacteria (18.3%), actinobacteria (15.5%) and alphaproteobacteria (13.1%, Fig. S7a), all commonly associated with soil and/or plant roots [7, 34]. No single bacterial OTU had a relative abundance above 2% in any sample. In contrast, fungal communities were dominated by one OTU (putatively identified as *Purpureocillium lilacinum*, syn: *Paecilomyces lilacinus*), which had a relative abundance between 49.9% and 66.1% in all samples (Fig. S7b).

For all bacterial and most fungal analyses, rarefied measures of microbial richness, inverse Simpsons diversity and Shannon diversity were significantly lower in root samples compared to samples from plant-free soil (Fig. S8; Table S4). Such reduction of microbial diversity in the rhizosphere has been reported previously [31, 35], and can be attributed to recruitment of specialised taxa that are better adapted to the rhizosphere environment. Furthermore, for all genotypes tested, crown root-associated communities had significantly lower diversity metrics than primary root-associated communities (Fig. S8). None of the richness and diversity metrics revealed statistically significant differences between *bx* mutant plants and WT plants (data not shown), indicating that *Bx* genes do not majorly affect the diversity of the maize rhizobiome.

Principal coordinate analysis (PCoA) was used to examine global differences in microbial community structure. Data were filtered to remove low abundance OTUs (see Supplementary Methods), generating a simplified data set of 3030 bacterial and 545 fungal OTUs. PCoA of bacterial OTUs on weighted UniFrac distances separated samples of plant-free soil from those of root+rhizosphere samples (components 1 and 2). In addition, the PCoA separated samples from primary and crown roots (components 2 and 3), but failed to separate samples from different *bx* genotypes (Fig. S9a and b). PCoA of fungal OTUs, using weighted Bray distances, did not result in separation by sample type (Fig. S9c and d). PERMANOVA revealed that the bacterial communities of root+rhizosphere samples differed significantly from those of plant-free control soil (*P* = 0.001; Table S5). Within the root+rhizosphere samples, there was a significant effect of root type (*P* = 0.002), no effect of plant genotype (*P* = 0.103), and a significant interaction between root type and plant genotype (*P* = 0.019; Table S5). Although the differences were less pronounced for the fungal OTUs, PERMANOVA analysis showed a statistically significant difference between samples from plant-free soil and the various root+rhizosphere combinations (*P* = 0.001; Table S5). In addition, root type showed a statistically significant effect on fungal OTU distribution (*P* = 0.010), but there was no effect of genotype (*P* = 0.121), nor was there a statistically significant interaction between the two (*P* = 0.440; Table S5). Together, the global analyses of OTU diversity and composition show that root presence and root type have stronger impacts on the microbial communities than *Bx* genotype.

### Identification of bacterial and fungal taxa that are influenced by *Bx* genotype

Bacterial and fungal OTUs that differed between samples were identified, using a generalised linear model that assumes a negative binomial distribution of OTUs and that corrects for increasing variance at lower OTU abundances [36]. Compared to plant-free soil, 545 bacterial OTUs were statistically enriched in root+rhizosphere samples, whereas 602 OTUs were depleted in one or more type of root samples. Of the fungal OTUs, only 61 were statistically enriched and 42 were statistically depleted in one or more type of root+rhizosphere samples. Thus, maize roots had a bigger effect on bacterial OTUs than fungal OTUs, supporting the global PCoA analysis (Fig. S9). Statistically altered OTUs are listed in Table S6 and graphically represented in Figs. S10 and S11). Root-enriched bacterial OTUs included members of the *Alphaproteobacteria*, *Betaproteobacteria, Gammaproteobacteria* and *Flavobacteriia* (Fig. S10), which are common in rhizosphere environments [7, 31, 37].

**Fig. 3:**
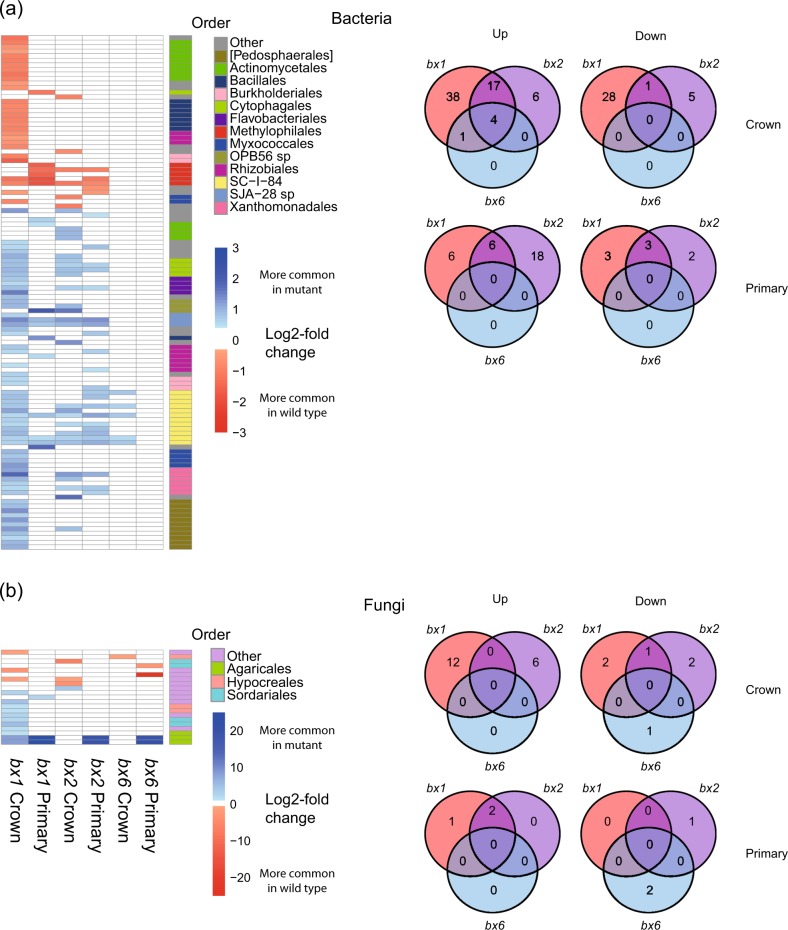
Differences in bacterial (**a**) and fungal (**b**) operational taxonomic units (OTUs) between roots from WT and *bx* mutant plants. Heat map projections on the left represent Log2 fold changes in relative OTU abundance between the *bx* mutant and the WT (crown and primary). Shown are statistically significant values only (*p* *≤* 0.05, corrected for false discovery). Red indicates *Bx*-stimulated OTUs with greater abundance in the WT than the *bx* mutant; shown in blue are *Bx*-repressed OTUs with greater abundance in the *bx* mutant than the WT. Note the difference in scale between bacterial and fungal OTUs. Phylogenies are shown at the order level for taxa that are represented more than twice. Venn diagrams on the right show numbers of unique and overlapping OTUs that are statistically different in relative abundance between *bx* mutant and WT roots (crown and primary). ‘Up’ and ‘Down’ refer to abundance in *bx* mutants relative to wildtype

To remove confounding effects of root type, statistical comparisons of root+rhizosphere samples between plant genotypes were performed separately for primary roots and crown roots. We identified a total of 113 bacterial and 21 fungal OTUs that were statistically altered in crown and/or primary roots by at least one *bx* mutation (Figs. 3 and S12, Table S7). Within this selection, the *bx1* and *bx2* mutations had the strongest effects on OTU abundances compared to the *bx6* mutation (Figs. 3 and S12). In crown roots, 89 and 33 bacterial OTUs were altered in the *bx1* and *bx2* mutant, respectively, of which 22 were shared between both mutant genotypes. In primary roots, only 12 and 24 bacterial OTUs were influenced by the *bx1* and *bx2* mutations, respectively, of which 9 were shared between both mutants (Fig. 3a). In crown roots, the *Bx1*-dependent and *Bx2*-dependent OTUs represented 10.3% and 4.1% of the sequence reads in the dataset, respectively; in primary roots *Bx1*-dependent and *Bx2*-dependent OTUs represented 14.8% and 4.5% of the sequence reads in the dataset, respectively. Thus, the impacts of the *Bx1* and *Bx2* genes in terms of numbers of bacterial taxa is greater for crown roots than for primary roots, but the relative abundances are comparable between both root types. Generally, members of the Proteobacteria (particularly beta-Proteobacteria) were responsive to *bx* mutations (either stimulated or repressed). Furthermore, the Verrucomicrobia and Bacteriodetes favoured roots of *bx* mutants, whereas Firmicutes and Actinobacteria favoured roots of WT plants.

In contrast to the bacterial OTUs, fewer fungal OTUs showed statistically significant differences in abundance between WT and *bx* mutants (Fig. 3b). The majority of *Bx*-responsive fungal OTUs (14) in crown roots were affected by the *bx1* mutation. Despite the relatively low numbers of *Bx*-dependent OTUs, their fold-changes were generally higher compared to the bacterial OTUs. Members of the Class Agaricomycetes and Sordariomycetes (Family Lasiosphaeriaceae) were particularly strongly affected by the *bx* mutations. *Bx*-dependent fungal OTUs included soil-borne pathogens, such as *Slopeiomyces cylindrosporus* (synonym *Gaeumannomyces cylindrosporus*, a pathogen of *Poaceae* and a relative of *Gaeumannomyces graminis* var. *tritici*, the causal agent of take-all disease in wheat [38, 39]), as well as *Ilyonectria macrodidyma* (synonym *Neonectria macrodidyma*), a causal agent of root rot [40].

### Relationship between *Bx*-dependent root chemistry and root microbiota

The UPLC-Q-TOF analysis of roots revealed a global impact of the *bx* mutations on root metabolism (Fig. 2). Accordingly, it is possible that the *Bx* biosynthesis genes influence the abundance of root-associated bacterial OTUs indirectly via BX-controlled metabolites. To address this hypothesis, we performed multiple correlation analysis between all *Bx*-dependent bacterial OTUs and *Bx*-dependent metabolite ions from crown and/or primary root samples. By selecting positive and negative correlations with coefficients ≥ 0.5 (Fig. S2), this analysis identified four different types of OTU–metabolite associations: (i) OTUs that are more abundant in the WT and correlate positively with root metabolites, (ii) OTUs that are more abundant in the WT and correlate negatively with root metabolites, (iii) OTUs that are more abundant in the *bx* mutants and correlate positively with root metabolites and (iv) OTUs that are more abundant in the *bx* mutants and correlate negatively with root metabolites.

**Fig. 4:**
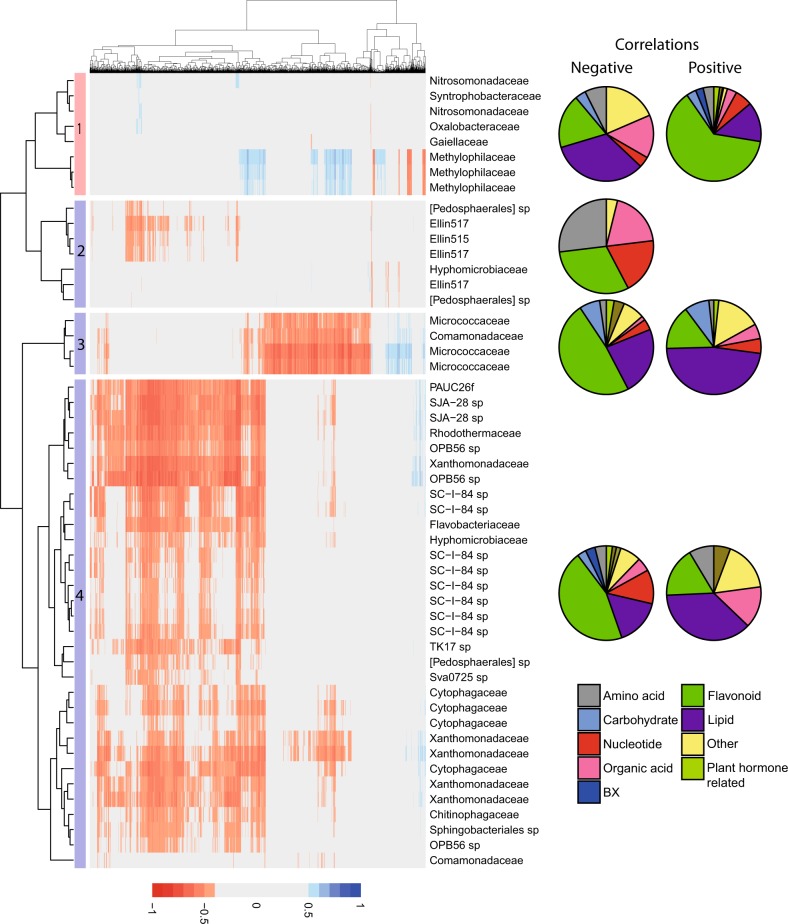
Spearman correlations between variance-stabilised relative OTU abundance and metabolite ions. Only correlations ≥ 0.5 are presented (positive correlations: blue; negative correlations: red). OTUs that are *Bx-*stimulated (enriched in the WT relative to the *bx* mutants) form one cluster (1). *Bx*-repressed OTUs (were enriched in *bx* mutants relative to WT) form three clusters (2–4). Pie charts show the distribution of putative metabolites between various metabolite pathways/classes for each cluster. The top eight correlations from each cluster are shown in Fig. S13

We identified eight BX-stimulated OTUs, which were enriched in WT roots compared to *bx1/2* mutant roots (Fig. 4, cluster 1). These OTUs showed positive correlations with 545 metabolites (association i) and negative correlations with 78 metabolites (association ii; Fig. 4). The strongest correlations were observed with three OTUs, all corresponding to members of the family Methylophilaceae, which can use methanol or methylamine as their sole carbon source [41]. Weaker and fewer correlations were observed with the remaining five OTUs, of which two belong to the Nitrosomonadaceae and one each to the Oxalobactereraceae, Syntrophobacteriaceae and Gaiellaceae. The putative identities of metabolites correlating with the cluster 1 OTUs are listed in Table S8. As expected for an untargeted metabolic analysis, putative assignments could only be made for ~50% of the ions. Consistent with *Bx*-stimulated OTUs correlating positively with metabolites from WT and *bx* mutant roots, BXs were strongly represented. However, positive correlations with other classes of metabolites were also prevalent. The most abundant metabolite class was the flavonoids, which contributed to more than half of the metabolites correlating positively with the *Bx*-stimulated OTUs. Considering that flavonoids can act as recruitment signals for beneficial soil bacteria [42], this result supports our hypothesis that the stimulatory effects of *Bx* genes on these bacterial OTUs could, in part, be mediated by BX-controlled plant metabolites, rather than BXs themselves.

In addition to the eight BX-stimulated OTUs, we identified 43 BX-repressed OTUs that were enriched in *bx* mutant roots compared to WT (Fig. 4). These BX-repressed OTUs showed positive correlations with 296 metabolites (association iii) and negative correlations with 1889 metabolites (association iv). All these correlations linked to three distinct OTU clusters (Fig. 4; clusters 2–4). The largest cluster (4) was dominated by negative correlations with metabolites. This cluster included members of the Xanthomonadaceae (a group that includes plant pathogens), *Adhaeribacter* sp. (within the *Cytophagaceae*, which can utilise cellulose as a growth substrate and adhere to surfaces), one member of the *Chitinophagaceae* (that can utilise chitin), OPB56, SC-I-84 and SJA-28 sp. The putative metabolites correlating negatively with these OTUs included BXs, flavonoids, lipids and nucleotides (Fig. 4; Table S8). A similar but less responsive cluster (2) of largely negative correlations contained four members of Ellin517, 2 *Pedosphaerales* sp. and a member of the *Hyphomicrobiaceae*. The final cluster (3) contained four OTUs, and was dominated by members of the *Micrococcaceae*. Interestingly, the correlations within this cluster largely mirrored that of cluster 1, indicating that conditions favouring the *Methylophilaceae* repressed the *Micrococcaceae* and vice versa.

## Discussion

The growing evidence for a functional contribution of root microbiota to plant growth and health has fuelled the desire to take advantage of the root microbiome in agricultural production systems [15, 43, 44]. However, this exploitation is hampered by a lack of understanding about the mechanisms driving rhizobiome assembly. Whilst it is often assumed that primary and secondary root metabolites play important regulatory roles, evidence for their importance is mostly based on studies of single microbial strains in relation to a single class of metabolites. For instance, it has previously been reported that the BX compound DIMBOA stimulates colonisation of maize roots by the plant-beneficial bacterial strain *P. putida* KT2440 [19]. Our study represents a global assessment of the influence of the BX biosynthesis pathway on the root metabolome and associated microbial communities. An unexpected outcome of the root metabolome analysis was that mutations in *Bx* biosynthesis genes caused major changes in root metabolites (Fig. 2). The *bx1* and *bx2* mutations, which almost completely blocked root BX biosynthesis (Fig. 1b, c), induced the strongest shifts in the root metabolome (Fig. 2 and S4). This indicates that BXs act as endogenous regulators of root metabolism, in addition to their previously characterised activities as biocidal defence compounds and semiochemicals [23]. Accordingly, the *bx1* and *bx2* mutations had the most significant impacts on rhizobiome composition of maize. In the most extreme case, 3% of bacterial OTUs, representing 10.3% of bacterial sequences in the dataset were statistically affected by the *bx1* mutation in crown roots. Since 31% of the bacterial OTUs, representing 25.2% of all bacterial sequences in the dataset, were directly influenced by the presence of crown roots, these results suggest that BXs are particularly important for rhizobiome assembly. As can be expected from the proximity of the *Bx1* and *Bx2* genes in the BX biosynthesis pathway (Fig. 1a), both mutations had comparable impacts on the root metabolome and associated bacterial communities (Figs. 2 and 3, respectively). By contrast, the *bx6* mutation had a relatively minor impact on the root metabolome (Fig. 2) and a weaker impact on bacterial OTUs than the *bx1* and *bx2* mutations (Fig. 3), which is likely due to the leaky nature of the *bx6* mutation [28].

The recent study by Hu et al. [25] identified the DIMBOA catabolite MBOA as a root-derived compound of maize that alters microbial soil communities. Plants growing on soil that had been conditioned by BX-producing maize or treated with MBOA showed increased defence signalling activity and enhanced resistance to herbivory. Hu et al. [25] concluded that the accumuation of MBOA by degradation of BXs from wild type roots conditions the soil for a resistance-inducing microbiome that protects plants of the next generation against herbivory. However, considering the stability of MBOA in soil [25, 45], it is equally possible that residues of MBOA induce changes in root metabolism that, in turn, recruit resistance-inducing microbiota. This alternative hypothesis is reconcilable with the finding by Hu et al. [25] that X-ray sterilisation of MBOA-conditioned soil eliminates its resistance-inducing activity [25], since MBOA-exposed roots would fail to recruit resistance-inducing microbiota from sterilised soil. Our conclusion that BXs act as regulators of a broad range of secondary root metabolites (Fig. 2), of which many correlate with specific clusters of taxonomically related rhizosphere OTUs (Fig. 4), supports this alternative hypothesis. Thus, in addition to a direct pathway by which root-exuded BXs recruit resistance-inducing rhizosphere microbes, we propose that BXs can also assemble a resistance-inducing rhizobiome indirectly, by acting as within-plant signalling metabolites that induce the production and release of a wider set of rhizosphere-active semiochemicals. Our findings suggest that genetic control of the rhizobiome is far more complicated than previously considered.

The simultaneous profiling of metabolic and microbial impacts by independent mutations in the same pathway represents a novel approach to potentially discover novel rhizosphere semiochemicals. Although some associations between metabolite classes and associated OTUs may be correlations without causative mechanisms, the emergence of distinct correlative patterns supports our notion that BXs influence root microbiota via regulation of other, rhizosphere-active, semiochemicals. The importance of flavonoids in rhizosphere interactions is well-characterised. Apart from their antimicrobial activities, they have been reported to act as chemo-attractants for nitrogen-fixing rhizobia in legumes, influence quorum sensing of other soil bacteria, and affect spore germination and branching of arbuscular mycorrhizal fungi [42, 46].

The three *Bx*-stimulated OTUs that showed the strongest and highest numbers of positive correlations with metabolite ions belonged to the *Methylophilaceae* (Fig. 4). We re-analysed the data presented in this manuscript in combination with that of Hu et al. [25], using a common analysis pipeline (Supplementary data file 1). Strikingly, despite the differences in genetic background of the *bx1* mutation, the soil, and growth conditions between the field experiment of Hu et al. [25] and our controlled environment experiment, the stimulatory effect of the *Bx1* WT roots on two Methylophilaceae OTUs was reproducible between both studies (Supplementary data file 1). These two OTUs also showed strong positive correlations in our study with root exudation of flavonoid metabolites (Fig. 4), reinforcing our notion that BX-controlled metabolites regulate root-associated microbes. This outcome makes Methylophilaceae indicators, and possible contributors, to plant health-promoting soil feedback responses, such as reported by Hu et al. [25]. Members of the Methylophilaceae can use methanol or methylamine as their sole carbon source and have been reported to influence plant growth [41]. A major source of plant-derived methanol is pectin methylesterase (PME) activity at the cell wall, which is known to increase during plant defence [47]. Interestingly, BXs have also been shown to regulate cell wall-based defences against fungi and aphids [17, 18]. Accordingly, it is possible that the regulatory function of BXs in cell wall defence extends to roots, where they increase PME activity to sustain populations of methanol-consuming and resistance-inducing methylotrophic bacteria.

In addition to the *Bx*-stimulated OTUs, we detected 43 *Bx*-repressed OTUs that correlated with root metabolite ions. These OTUs included members of the Adhaeribacter (*Cytophagaceae*), *Xanthomonadaceae*, SC-I-84 and SJA-28 taxa, all of which have been reported to be present in soil [48–51]. Furthermore, plant roots have been shown to select against soil-inhabiting SJA-28 bacteria [52]. Accordingly, the lack of BXs in the *bx1* and *bx2* mutant may have compromised their ability to restrict root colonisation by SJA-28 bacteria. A similar situation could apply to other members of the *Bx*-repressed OTUs in our study, such as the *Xanthomonadaceae*. Members of this family can infect plants through immune-suppressing effector proteins [53], supporting the hypothesis that BXs counteract the development of pathogenic microbes in the rhizosphere. Neal et al. [19] reported previously that DIMBOA inhibits growth of the soil-borne pathogen *Agrobacterium tumefaciens*, while resistance-inducing *P. putida* bacteria were found to be tolerant to this root-exuded BX [19]. Together with the recent results of Hu et al. [25], who showed that BX-producing maize conditions soils for resistance-inducing activity [25], these data collectively support the notion that root production of BXs stimulates the formation of a plant health-promoting soil microbiome.

Previous studies about the impacts of individual genes on rhizosphere communities are predominantly based on Arabidopsis and *Lotus japonicus* [14, 27, 54]. Although these studies have provided important insights into the genetic control of root-associated microbial communities, the root systems of these model dicot plant species are very different to cereal root systems [55]. Our results show that genetic control of both the metabolome and microbiome in maize varies between different root types, which are not present in Arabidopsis or *L. japonicus*. Thus, previous studies may have under-estimated the complexity of the mechanisms that shape rhizosphere communities. In that context, our study makes an important contribution towards the development of a crop-based model system for rhizosphere biology. Our results also illustrate the importance of considering multiple kingdoms/domains of microbes. Root-associated fungi are thought to be extremely diverse and important to plant health and ecosystem processes [56, 57], yet the majority of microbial rhizosphere studies focus on prokaryotes only [58, 59]. Our study is the first to simultaneously characterise impacts of different mutations within the same metabolic pathway on root-associated bacterial and fungal communities. In agreement with the recent study by Hu et al. [25], we found that mutations in *Bx1* had greater impacts on bacterial communities than on fungal communities. It is plausible that the dominant fungi in the soil communities tested were mostly filamentous saprophytes, such as *P. lilacinum* [60] and members of Mortierellales (Fig. S7b [61]), which are less likely to be reliant on, and influenced by, root-derived chemicals than bacteria. This is consistent with other studies, reporting overall weaker rhizosphere effects for fungi than bacteria [58, 62]). Nonetheless, we found that selected fungi are affected by mutations in the BX pathway, including phytopathogenic fungi, such as *S. cylindrosporus* and *I. macrodidyma*. This further supports the notion that BXs suppress soil-borne diseases. Evidently, more research is needed to address the extent and exact contribution of root-produced BXs to the suppression of soil-borne pathogens, and whether there are undesirable side-effects, such as attraction of specialised root herbivores [20]. Depending on the outcome of such studies, future breeding programmes for increased root BX production could make a contribution to better control of soil-borne diseases.

By studying the impacts of independent mutations in the BX biosynthesis pathway on both root metabolism and microbial communities, we have generated new insights into the factors shaping the maize rhizobiome. We have shown that the effects of *Bx* genes vary according to root type and position in the pathway, influencing bacterial and fungal communities to different extents. Moreover, we have provided plausible evidence that *Bx* genes influence rhizobiome communities via endogenous regulatory activity on a wider spectrum of plant-derived rhizosphere signals, including flavonoids. As such, our study supports the growing notion that BXs represent important signalling molecules in below-ground plant–biotic interactions. Moreover, the experimental strategy outlined in this paper represents a novel approach to generate new hypotheses and tools to study the effects of the root rhizobiome on plant performance.

## Supplementary information

Supplementary methods

Supplementary figures

Supplementary data file 1

Supplementary Tables
