# Supplementary material for: Metabolic regulation of the maize rhizobiome by benzoxazinoids
Source: ISME J. 2019 Feb 22;13(7):1647–58. doi: 10.1038/s41396-019-0375-2 (PMC6592824; doi:10.1038/s41396-019-0375-2)
Supplement: Supplementary file 1 — Supplementary methods [file 41396_2019_375_MOESM1_ESM.docx]

**Supplementary methods**

**Plants, growth substrate and growth conditions.**

Seeds were surface-sterilised by exposing them to chlorine gas for 3h (generated by mixing 100 ml bleach and 5 ml concentrated HCl in a beaker). Seeds were then imbibed overnight in sterile H_2_O and placed on damp, sterile filter paper in the dark at 23-25°C for two days to germinate. The growth substrate consisted of a 3:1 (v:v) mixture of agricultural soil: autoclaved perlite. Agricultural soil was collected from an arable field at Spen farm, Leeds, UK (53.874944, -1.320165), sieved to 4.75 mm and air-dried. The mean annual temperature at the field site is 9.2 ^o^C and mean annual precipitation is 674 mm [1]). In the year of sampling the field was used to grow winter barley and the previous year, winter wheat. The soil is a silt loam in the Aberford series of Calcaric Endoleptic Cambisols underlain by dolomitic limestone of the Cadeby formation (British Geological Survey, 2018). The C and N content of the soil used in the experiment were quantified as described previously [1]. The soil pH was 7.7, a total nitrogen (N) content of 0.21% and a total carbon (C) content of 2.89%, of which 1.97% was organic carbon by mass. Pre-germinated seeds were planted (1 seed/ 750ml pot) into the growth substrate (~ 1.5 cm depth) and 300 ml distilled H_2_O added to each pot to bring the soil to field capacity. Growth substrate was covered with a layer of black plastic beads (4mm diameter) to prevent algal growth. Pots were left to drain then placed on individual saucers. No additional fertiliser was added to the pots. Plants were grown in a growth chamber with the following conditions: 16/8 h light/dark at 20°C with an average irradiance at pot height of 140 µmol m^-2^ s^-1^ and a relative humidity (RH) of 60%.

All plants used for these experiments were genotyped by PCR, using primers designed to anneal to the WT *Bx* genes and/or the mutated transposon-inserted *bx* gene. Plants used for UPLC-Q-TOF analysis were genotyped by DNA extracted from freeze-dried shoots, as described by [2]. Plants used for microbiome analysis were genotyped by DNA extracted from the crown roots as described in the main Materials and Methods section (microbial community profiling). PCR reactions were performed with 0.5 µl of extracted DNA, 0.5U MyTaq™ DNA Polymerase (Bioline, London, UK) and 0.4 µM of primers in the presence of the manufacturer’s buffer in a reaction volume of 10 µl. PCR conditions were 94^o^C for 5 min, 35 cycles of 94^o^C for 30s, 57^o^C for 30s and 72^o^C for 30 s with a final 5 min extension at 72°C. 5µl of each PCR reaction were checked by gel electrophoresis. Primers used are detailed in Table S1a.

**Untargeted root metabolite profiling by UPLC-Q-TOF analysis.** Crown roots and primary roots [3] were collected, flash frozen in liquid nitrogen and freeze dried. For each genotype/tissue combination, root samples from six independent plants were sampled. Extraction of root metabolites was performed as described in ref [4]. Briefly, metabolites were extracted twice from freeze-dried material (10 mg) in ice-cold extraction buffer (methanol: water: formic acid, 95: 4.9: 0.1 v:v:v), and subjected to untargeted metabolic profiling analysis, using an ACQUITY ultra-high-pressure liquid chromatography (UPLC) coupled to a SYNAPT G2 Q-TOF mass spectrometer with an electrospray (ESI) ionization source (Waters, UK). Chromatographic separation occurred at a flow rate of 0.4 mL min^−1^, using an ACQUITY UPLC BEH C18 column (2.1 × 50 mm, 1.7 μm, Waters) coupled to a C18 VanGuard column. Runs in negative (ESI^-^) and positive (ESI^+^) ionisation mode were separated by two successive injections with 50% methanol (v:v) to allow for stabilization of the ion source. Ions were detected over a mass range of 50 – 1200 Da (scan time 0.2 sec.), using full MS scans. A MS^E^ function was applied by ramping the collision energy in the transfer cell from 5 to 45 eV. Specific ESI- and ESI+ settings were as described previously [5]. The entire system was controlled by MassLynx v 4.1 software.

For unsupervised Principle Component Analysis (PCA) of UPLC-Q-TOF data, raw peaks were aligned and integrated by XCMS R (v. 3.1.3; Smith *et al.*, 2006). Peaks were retained for analysis when present in all replicates at a threshold intensity (*I*) of 10 and at maximum resolution range of 20 ppm. Values from each run were normalised to total ion current (TIC) and corrected for dry weight. Pooled data from ESI^+^ and ESI^-^ were median-normalised, cube-root-transformed and Pareto-scaled prior to PCA, using MetaboAnalyst (v. 3.0; http://www.metaboanalyst.ca; [7]. Ions that differed significantly in abundance between genotypes for a given root type (crown or primary) were identified using a Generalised Linear Model (implemented in DESeq2), using a ‘local’ distribution model of variance with ion intensity and corrected for false discovery by the Wald method.

**Microbial community profiling.**

All samples were ground to a fine powder, using liquid nitrogen in a pestle and mortar (baked at 300 ^o^C between samples). DNA was extracted from approximately 0.25 g of the homogenised powder using a PowerSoil® DNA Isolation kit (MO BIO Laboratories, USA), according to the manufacturer’s instructions. Quantification of total bacterial and fungal DNA was based on quantitative PCR, using a Rotor-Q machine (Qiagen). For quantification of bacterial DNA, samples were compared to standards of genomic DNA from *Escherichia coli* strain B (Sigma-Aldrich), using primers against 16S rRNA gene sequences (799F and 1193R; Table S1b). For quantification of fungal DNA, samples were compared to standards of genomic DNA from *Candida tropicalis* (Castellani) Berkhout (ATCC, Teddington, UK), using primers against ITS rRNA sequences (fITS7 and ITS4; Table S1b). Reactions contained 5 μl SYBR Green PCR master mix (QIAGEN, Netherlands), 2 μl of 1.25 μM primers and 2 μl of the target DNA sample in a total volume of 10 μl, with an initial denaturation of 95 ^o^C for 10 seconds followed by 40 cycles of 95 ^o^C for 10 seconds and 60 ^o^C for 45 seconds. The specificity of all primers was verified by assessing melting curves of PCR products. A standard curve was created by plotting the sample-specific Ct (take-off cycle) against the standard DNA concentration, where Ct was defined as the cycle in which the increase in fluorescence was 20% of the peak increase in fluorescence (QIAGEN 2012). The amount of DNA in each sample was then corrected for mass of material from which the DNA was extracted. The amount of DNA per g of sample was expressed in relative terms compared to WT samples.

Illumina MiSeq amplicon sequencing was used to describe the bacterial and fungal microbial communities from root and soil samples. Amplification of the V5-V7 region of bacterial 16S rRNA gene fragments was performed, using primers 799F and 1193R [8, 9], modified to include the Illumina overhang adapter nucleotide sequences, as described in [5]. PCR reactions were performed with 0.8 µl of 1/2 concentration DNA extracts, 2U MyTaq™ DNA Polymerase (Bioline, London, UK) and 0.4 µM of primers in the presence of the manufacturer’s buffer in a reaction volume of 20 µl. Each reaction was performed in triplicate. PCR conditions were: 94^o^C for 2 min, 27 cycles of 94^o^C for 30s, 55^o^C for 30s and 72^o^C for 30 s with a final 1 min extension at 72°C. Five µl of each PCR reaction was used to verify PCR products by gel electrophoresis, and 8µl of each of the three PCR reactions from each sample were pooled. PCR products of c. 500bp in the pooled amplicons were then cleaned by gel extraction using a 1.2% agarose gel and QIAquick Gel Extraction Kit (Qiagen), following the manufacturer's instructions to remove larger (approx. 750bp) amplification products derived from maize mitochondrial DNA [8]. Partial 5.8S, ITS2 and partial 28S fungal fragments were amplified, as described above, but using 0.8µl of undiluted DNA extract as the template, and primers based on fITS7 [10] and ITS4 [11], with an annealing temperature of 57°C and 30 cycles. Five µl of the purified amplicons were indexed, using a Nextera XT Index v2 kit (Illumina Inc., USA) by amplification with 1 unit of KAPA HiFi HotStart DNA polymerase (Kapa Biosystems, UK), 10 µl of 5x KAPA HiFi buffer, 0.5 mM MgCl_2_ and 5 µl of each index in a total reaction volume of 50 µl. PCR conditions were: 95^o^C for 3 min, 8 cycles of 95^o^C for 30s, 55^o^C for 30s and 72^o^C for 30s with a final 5 min extension at 72°C. Amplicons were checked by gel electrophoresis and then purified, using AMPure XP beads (Beckman Coulter Inc., USA). DNA quantities of indexed amplicons were quantified using PicoGreen (ThermoFisher), after which samples were pooled to a final concentration of 40 nM DNA. After attachment of dual indexes, quantification and pooling, amplicons were sequenced using a paired end 2 x 250 bp cycle kit on a MiSeq machine running v2 chemistry (Illumina Inc, at the Earlham Institute, Norwich, UK). Full details of amplification and sequencing are provided in the supplementary methods. Sequences have been deposited in the European Nucleotide Archive under accession number PRJEB24367. The numbers of reads obtained for each sample are shown in Table S3.

De-multiplexed FastQ files were filtered using USEARCH 9.1 with a maxEE value of 1 [12]. Bacterial 16S rRNA sequences were paired whereas for fungal ITS sequences only forward reads were analysed due to longer and more variable amplicon lengths. Primer sequences were removed and chimeras were detected using UCHIME [13], using both reference based (RDB v16) and *de novo* detection methods. Subsequent analysis was conducted with QIIME [14]. For 16S rRNA sequences, Operational Taxonomic Units (OTUs) were defined by clustering using UCLUST (97% similarity; [12] and taxonomies assigned by alignment to the Greengenes database (v13.8), using PyNAST [15]. For analysis of ITS sequences, comparisons were made to the QIIME/UNITE reference ITS database (v7.1). Tree construction was omitted for fungal data as the ITS region is not phylogenetically informative. Singletons were removed from both data sets as these are typically sequencing errors [16].

Further analysis was conducted in R, using phyloseq [17]. Richness and diversity indices (Shannon and Inverse Simpson) measurements were calculated by rarefying the data (to 52,176 reads for prokaryotic samples, 45,382 reads for fungal samples) 100 times. Sequences were filtered to retain those obtained at least 3 times in 20% or more of the samples. Read counts were normalised to 100%. Ordinations were performed by Principal Coordinates Analysis (PCoA) using weighted phylogenetically-aware Unifrac distance measures for prokaryotic sequences [18] or Bray-Curtis distance measures for fungal data. PERMANOVA analysis with 999 permutations was used to statistically test for compositional differences between samples [19]. Differences in relative abundances were analysed using a Generalised Linear Model implemented in DESeq2 [20], assuming a ‘parametric’ negative log normal distribution of variance with OTU abundance and correction for false discovery (Wald method).

**REFERENCES**

1. Holden J, Grayson RP, Berdeni D, Bird S, Chapman PJ, Edmondson JL, et al. The role of hedgerows in soil functioning within agricultural landscapes. *Agriculture, Ecosystems & Environment* 2019; **273**: 1–12.

2. Edwards K, Johnstone C, Thompson C. A simple and rapid method for the preparation of plant genomic DNA for PCR analysis. *Nucleic Acids Res* 1991; **19**: 1349.

3. Hochholdinger F, Tuberosa R. Genetic and genomic dissection of maize root development and architecture. *Current Opinion in Plant Biology* 2009; **12**: 172–177.

4. Pétriacq P, Ton J, Patrit O, Tcherkez G, Gakière B. NAD Acts as an Integral Regulator of Multiple Defense Layers. *Plant Physiol* 2016; **172**: 1465–1479.

5. Pétriacq P, Williams A, Cotton A, McFarlane AE, Rolfe SA, Ton J. Metabolite profiling of non-sterile rhizosphere soil. *Plant J* 2017; **92**: 147–162.

6. Smith CA, Want EJ, O’Maille G, Abagyan R, Siuzdak G. XCMS: processing mass spectrometry data for metabolite profiling using nonlinear peak alignment, matching, and identification. *Anal Chem* 2006; **78**: 779–787.

7. Xia J, Sinelnikov I V., Han B, Wishart DS. MetaboAnalyst 3.0-making metabolomics more meaningful. *Nucleic Acids Research* 2015; **43**: 251–257.

8. Bodenhausen N, Horton MW, Bergelson J. Bacterial Communities Associated with the Leaves and the Roots of Arabidopsis thaliana. *PLOS ONE* 2013; **8**: e56329.

9. Chelius MK, Triplett EW. The Diversity of Archaea and Bacteria in Association with the Roots of Zea mays L. *Microbial Ecology* 2001; **41**: 252–263.

10. Ihrmark K, Bödeker ITM, Cruz-Martinez K, Friberg H, Kubartova A, Schenck J, et al. New primers to amplify the fungal ITS2 region – evaluation by 454-sequencing of artificial and natural communities. *FEMS Microbiol Ecol* 2012; **82**: 666–677.

11. White TJ, Bruns T, Lee S, Taylor JW. Amplification and direct sequencing of fungal ribosomal RNA genes for phylogenetics. *PCR Protocols: A Guide to Methods and Applications*. 1990. Academic Press, Inc, pp 315–322.

12. Edgar RC. Search and clustering orders of magnitude faster than BLAST. *Bioinformatics* 2010; **26**: 2460–2461.

13. Edgar RC, Haas BJ, Clemente JC, Quince C, Knight R. UCHIME improves sensitivity and speed of chimera detection. *Bioinformatics* 2011; **27**: 2194–2200.

14. Caporaso JG, Kuczynski J, Stombaugh J, Bittinger K, Bushman FD, Costello EK, et al. QIIME allows analysis of high-throughput community sequencing data. *Nat Methods* 2010; **7**: 335–336.

15. Caporaso JG, Bittinger K, Bushman FD, DeSantis TZ, Andersen GL, Knight R. PyNAST: a flexible tool for aligning sequences to a template alignment. *Bioinformatics* 2010; **26**: 266–267.

16. Majaneva M, Hyytiäinen K, Varvio SL, Nagai S, Blomster J. Bioinformatic Amplicon Read Processing Strategies Strongly Affect Eukaryotic Diversity and the Taxonomic Composition of Communities. *PLoS One* 2015; **10**.

17. McMurdie PJ, Holmes S. phyloseq: An R Package for Reproducible Interactive Analysis and Graphics of Microbiome Census Data. *PLoS One* 2013; **8**.

18. Lozupone C, Lladser ME, Knights D, Stombaugh J, Knight R. UniFrac: an effective distance metric for microbial community comparison. *ISME J* 2011; **5**: 169–172.

19. Wang Y, Naumann U, Wright ST, Warton DI. mvabund– an R package for model‐based analysis of multivariate abundance data. *Methods in Ecology and Evolution* 2012; **3**: 471–474.

20. Love MI, Huber W, Anders S. Moderated estimation of fold change and dispersion for RNA-seq data with DESeq2. *Genome Biol* 2014; **15**: 550.
