# Supplementary material for: Metabolic regulation of the maize rhizobiome by benzoxazinoids
Source: ISME J. 2019 Feb 22;13(7):1647–58. doi: 10.1038/s41396-019-0375-2 (PMC6592824; doi:10.1038/s41396-019-0375-2)

**Figure S1:** PCR confirmation of *bx* mutant genotypes by PCR, using primers specific for (**a**) WT *Bx1* gene, (**b**) mutant *bx1* gene (**c**) WT *Bx2* gene, (**d**) mutant *bx2* gene, (**e**) WT *Bx6* gene, and (**f**) mutant *bx6* gene. Lines above samples indicate the predicted genotype of the seeds tested. Molecular weight markers are 100 bp ladders. +/- indicate positive and negative PCR controls, respectively. Individual plants used in the experiments were verified for genotype for of all six genes, but only a sub-sample are shown.

**
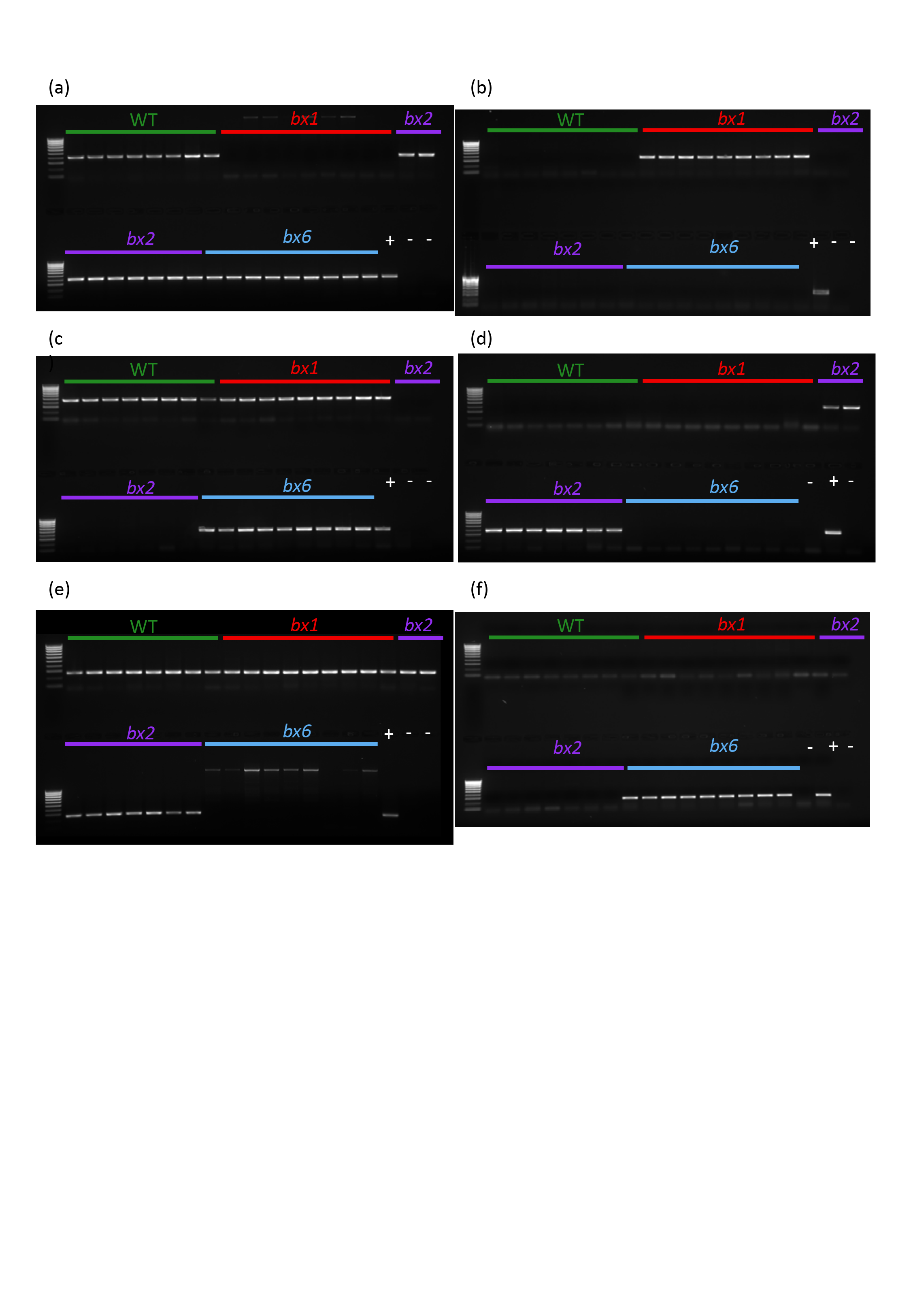
**

**Figure S2:** Flow chart of the multiple correlation analysis between *Bx*-dependent metabolite ions and bacterial OTUs. Negative numbers represent *Bx*-stimulated ions/OTUs that are statistically higher in samples from WT roots than *bx* mutant roots; positive numbers represent *Bx*-repressed ions/OTUs that are statistically higher in samples from *bx* mutant roots than WT roots.


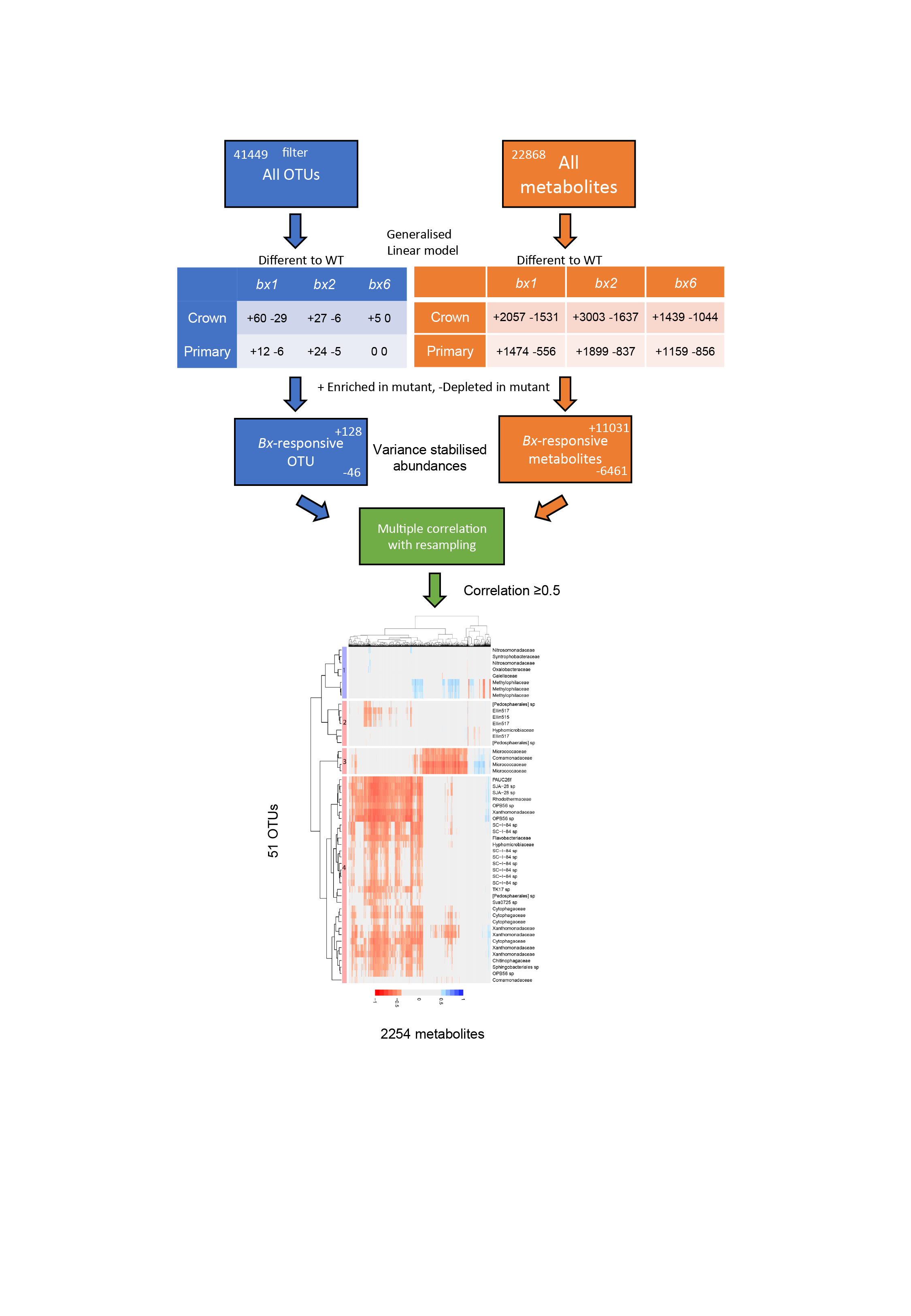


**Figure S3:** Representative growth phenotypes and masses of WT and *bx* mutant plants. (**a**) Photograph of plants at 17 days after planting. Dry masses of (**b**) shoots and **(c)** crown and primary roots of WT and *bx* mutant plants. **(d)** the relative proportion of root types sampled from WT and *bx* mutant plants. Crown (shaded bars) and primary (open bars) roots were distinguished on the basis of their position originating from the seed. Any roots which became detached from the seed during sampling are not included in these data. Data are means (±SE ; n = 10 except for *bx2* where n=9). There were no significant differences between WT and mutant plants (p > 0.05).

**
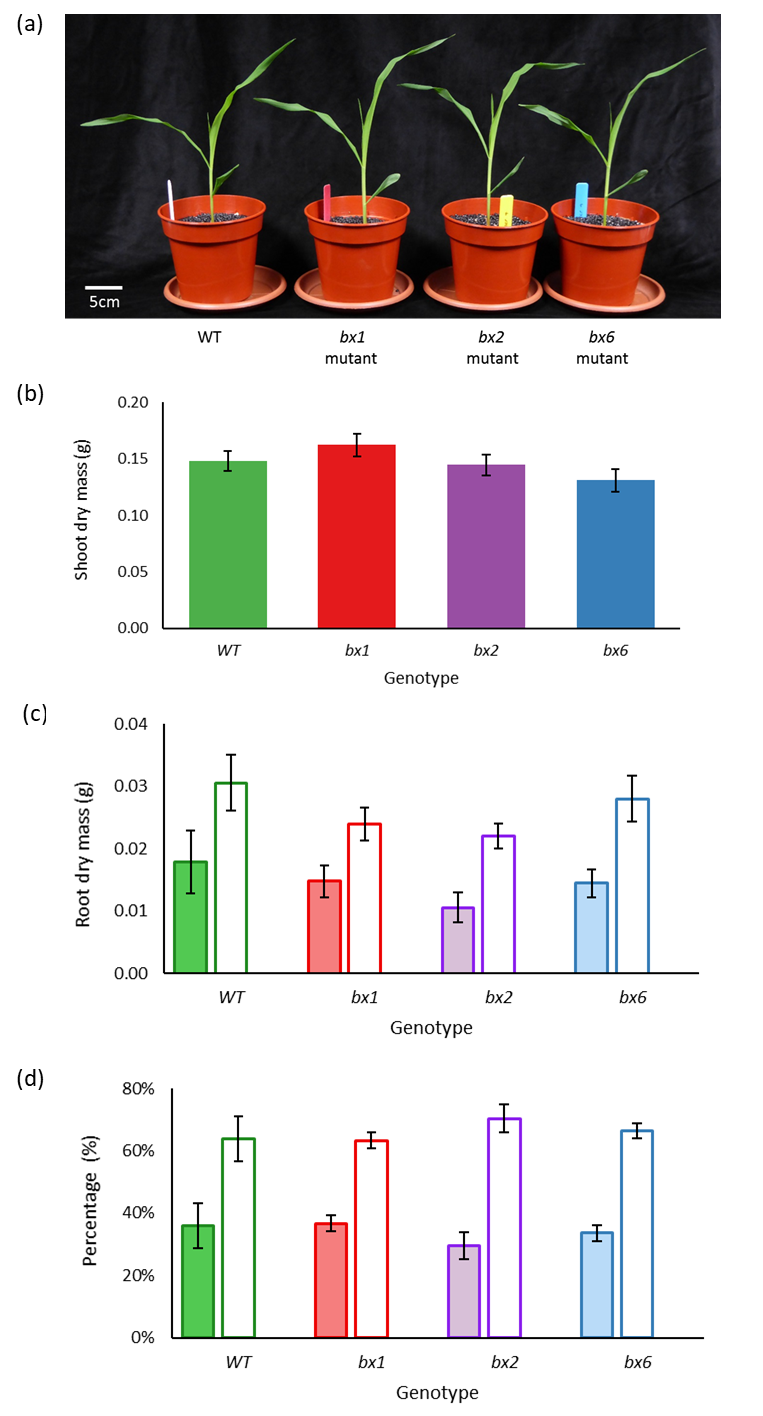
**

**Figure S4.** Statistical significance, fold-change and overlap between differentially expressed metabolite ions in *bx* mutant roots compared to WT roots (crown and primary). (**a**) Plots show statistical significance (y-axis) against fold-change (x-axis) for all ions considered in the analysis. Red symbols indicate statistically significant ions (*p*-adjusted < 0.05). Numbers in the margins of each plot represent statistically significant ions that fall beyond the axis range. (**b**) Venn diagrams show numbers of unique and overlapping OTUs that are statistically different in relative abundance between *bx* mutant(s) and the WT (crown and primary).


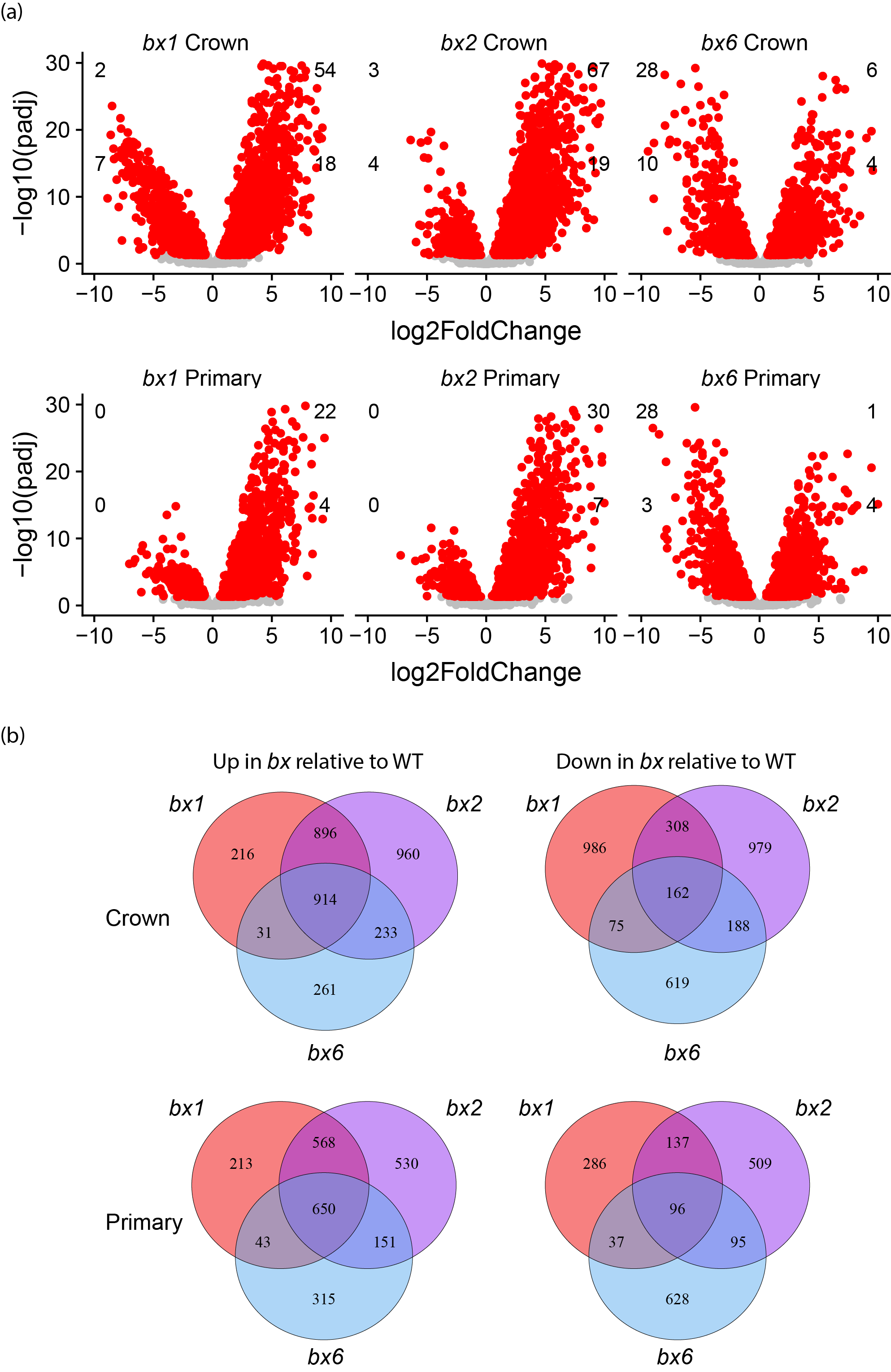


**Figure S5:** Quantification of total (a) bacterial and (b) fungal DNA by quantitative PCR of 16S and ITS rRNA gene sequences, respectively. Results are expressed relative to WT crown roots. There were no significant differences between WT and mutant samples (p>0.05).


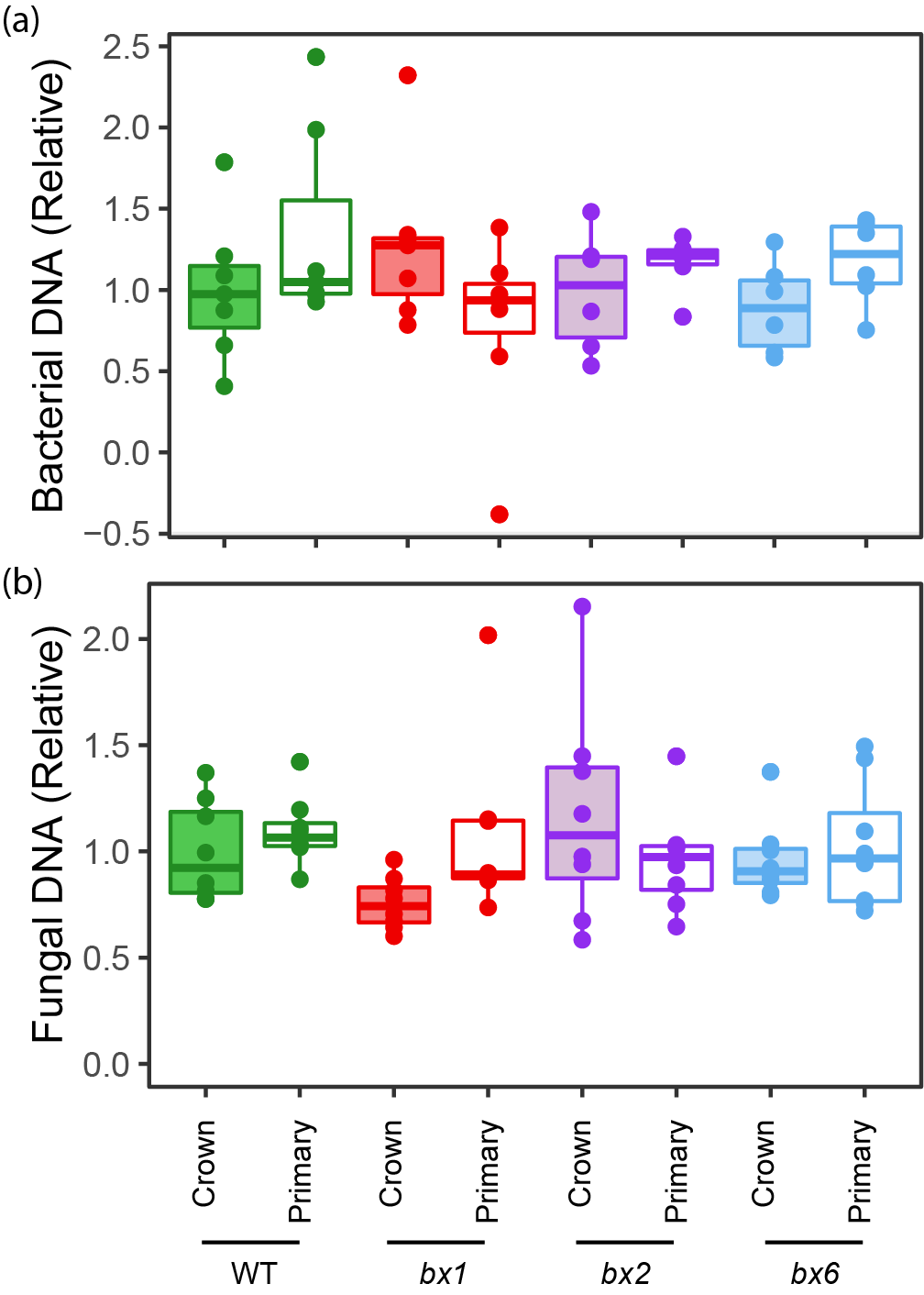


**Figure S6**: Rarefaction curves of (**a**) bacterial 16S rRNA OTUs and (**b**) fungal ITS OTUs. Curves were generated after singleton removal and each data point represents the average of 8 samples. Symbols represent soil types (triangles= plant-free control soil, square= primary roots, circle= crown roots); colours represent genotypes (green = WT, red = *bx1*, purple = *bx2*, blue = *bx6*).


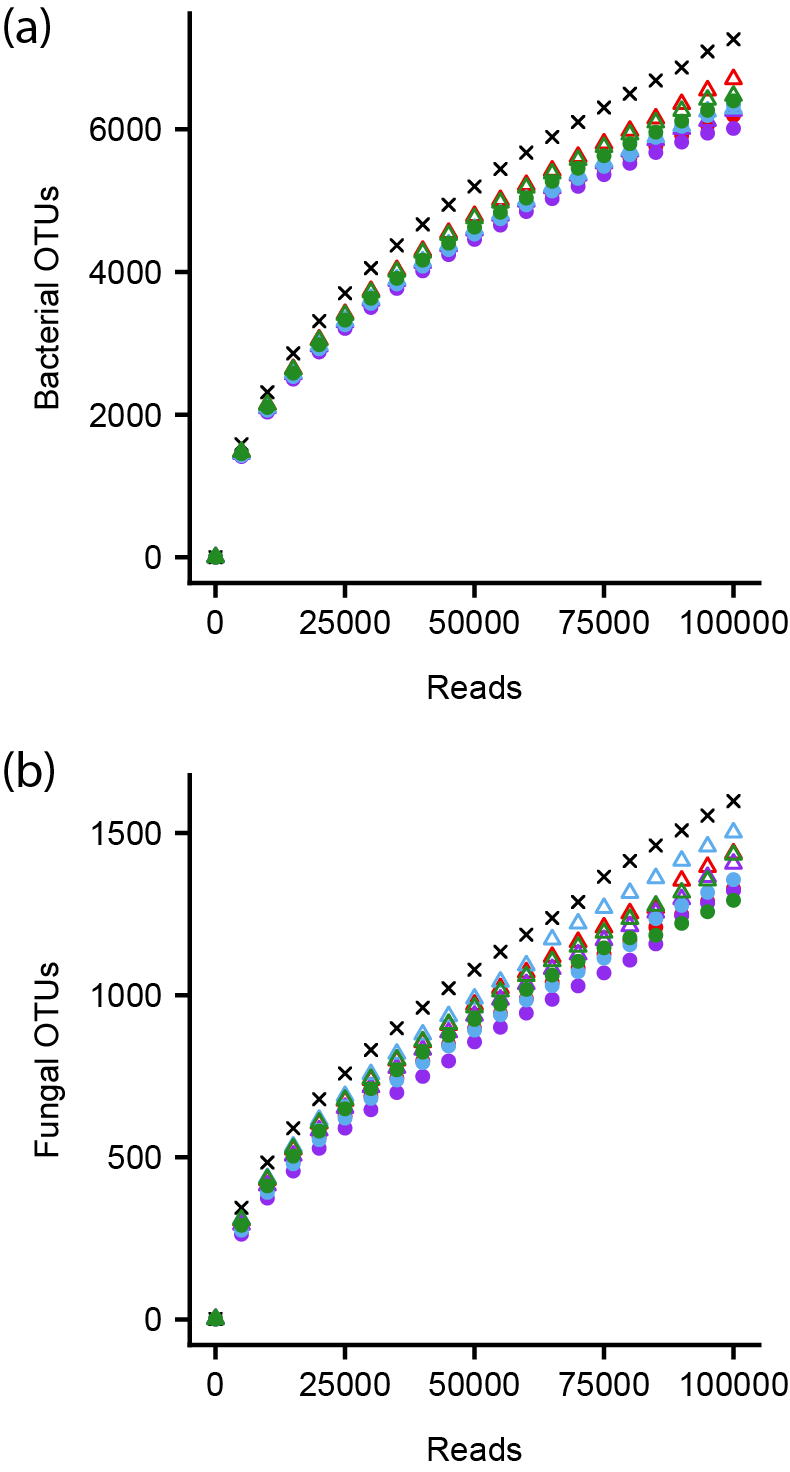


**Figure S7.** Relative abundances of (**a**) bacterial OTUs at the Class level and (**b**) fungal OTUs at the order level. Values sown are means (n=8) corrected for sampling depth. OTUs that represent <1% of the total are annotated as ‘Other’.


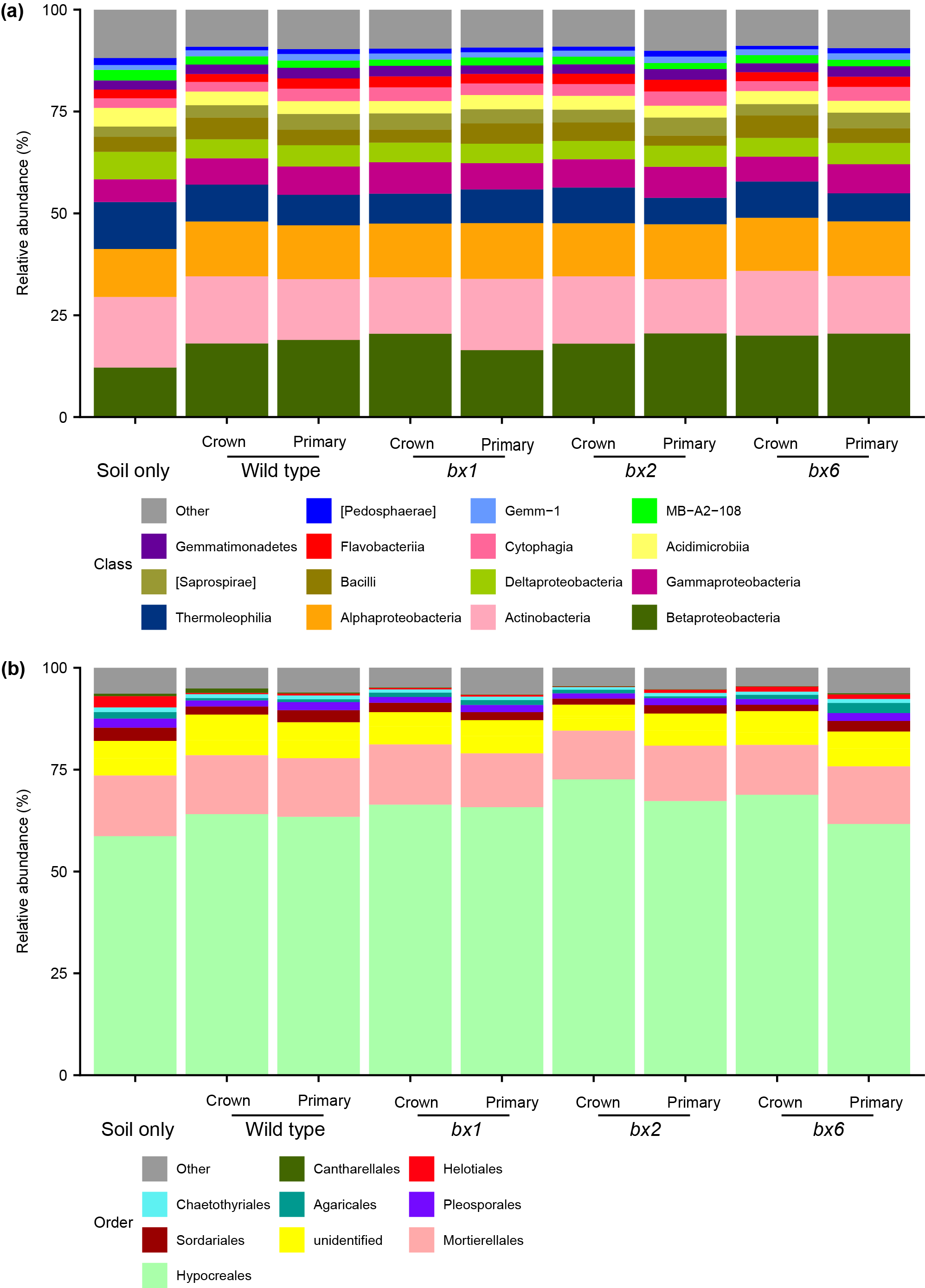


**Figure S8.** Diversity metrics of (**a**) bacterial communities from 16S rRNA gene data sets and (**b**) fungal communities from ITS data sets. Metrics were based on 8 independent biological replicates (n=8).


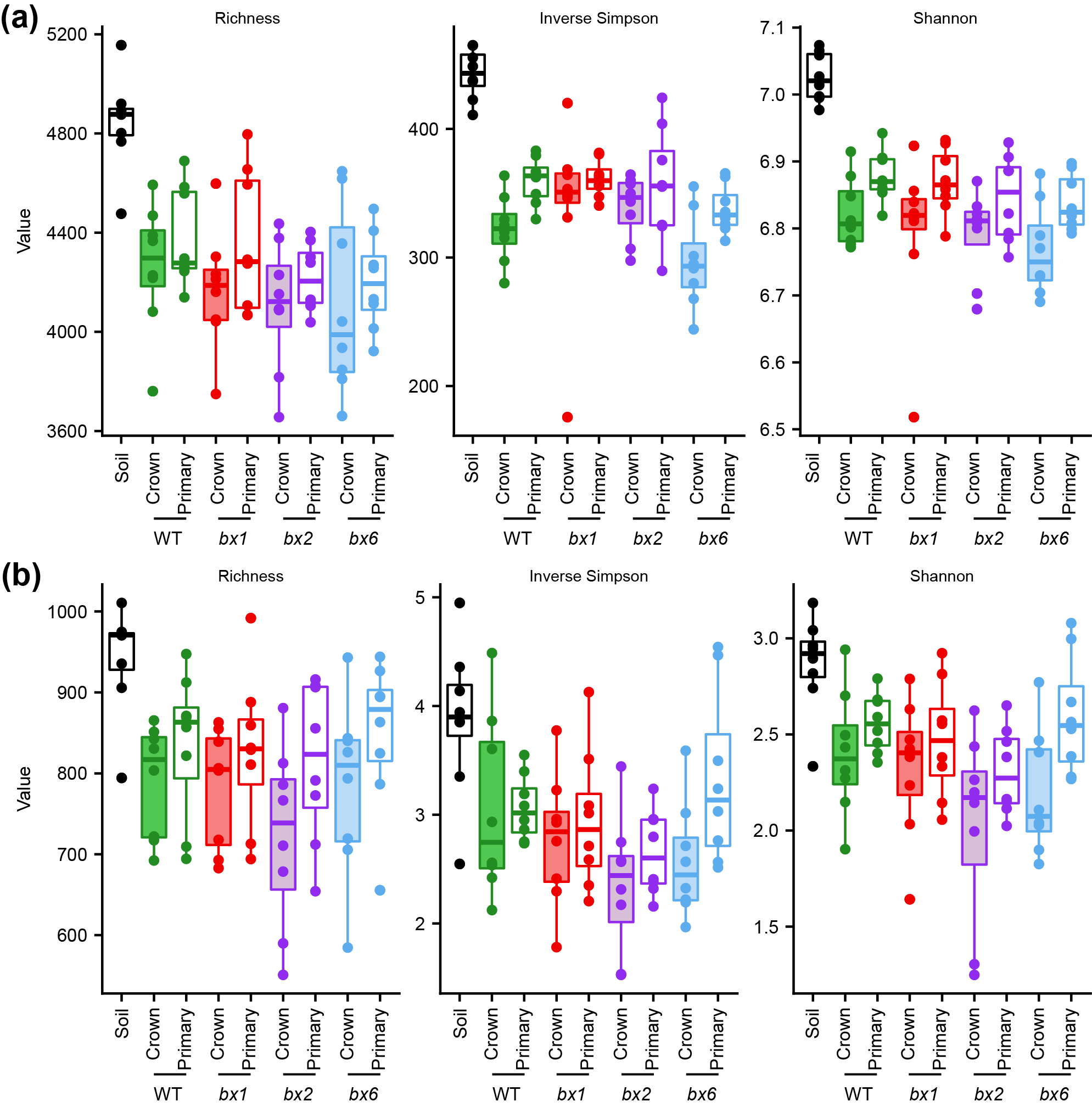


**Figure S9.** Principal Coordinate Analysis (PCoA) of bacterial 16S rRNA sequences based on weighted Unifrac distances **(a-b),** and fungal ITS sequences based on Bray-Curtis distances **(c-d)**. Shown are components 1 *vs* 2 (**a**, **c**) and 2 *vs* 3 (**a**, **c**). For bacterial samples, circles represent confidence intervals (omitted from fungal plots for clarity). Symbols represent sample soil/root types (crosses= plant-free control soil, closed circles= primary roots, open triangles= crown roots). Colours represent genotypes (green = WT, red = *bx1*, purple = *bx2*, blue = *bx6*).


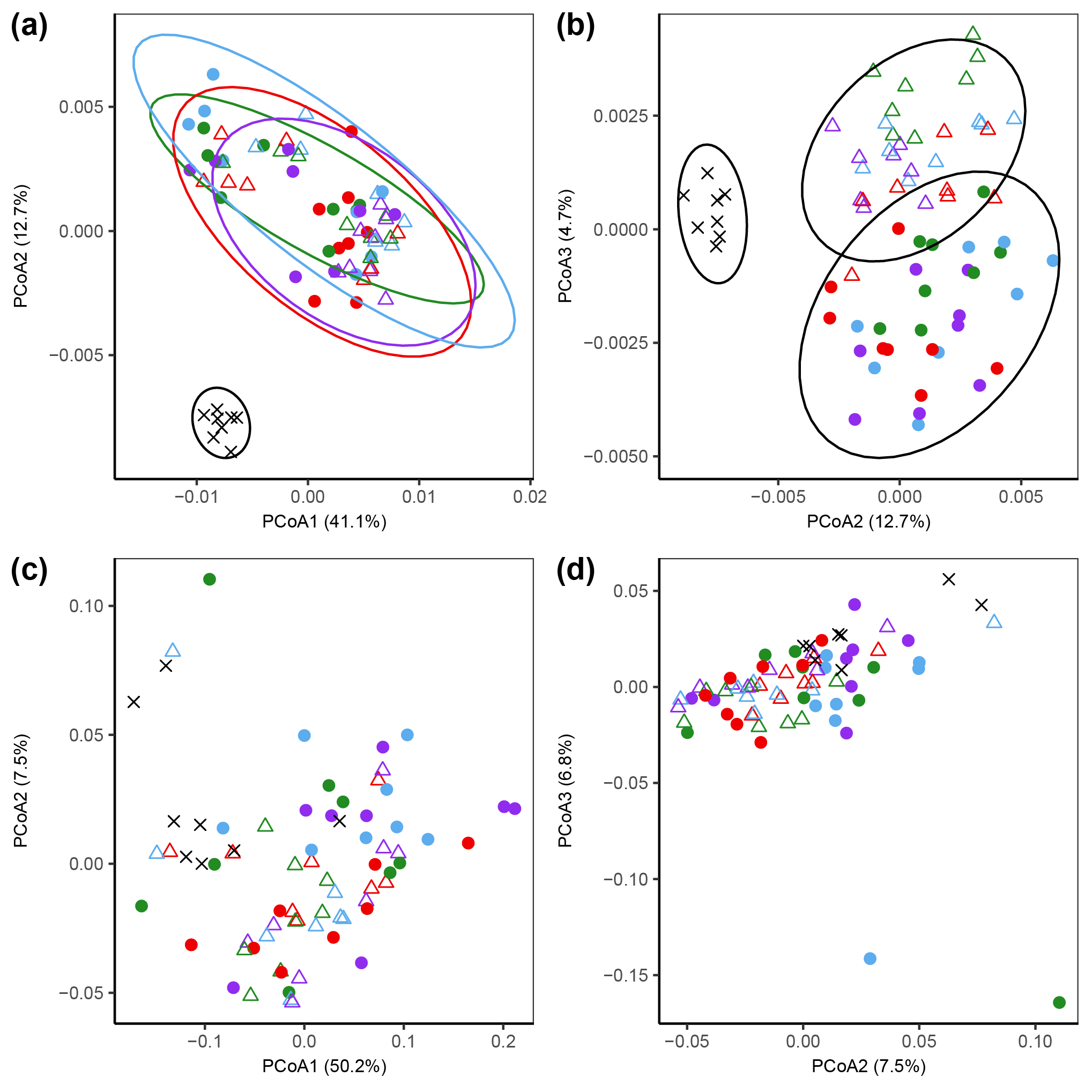


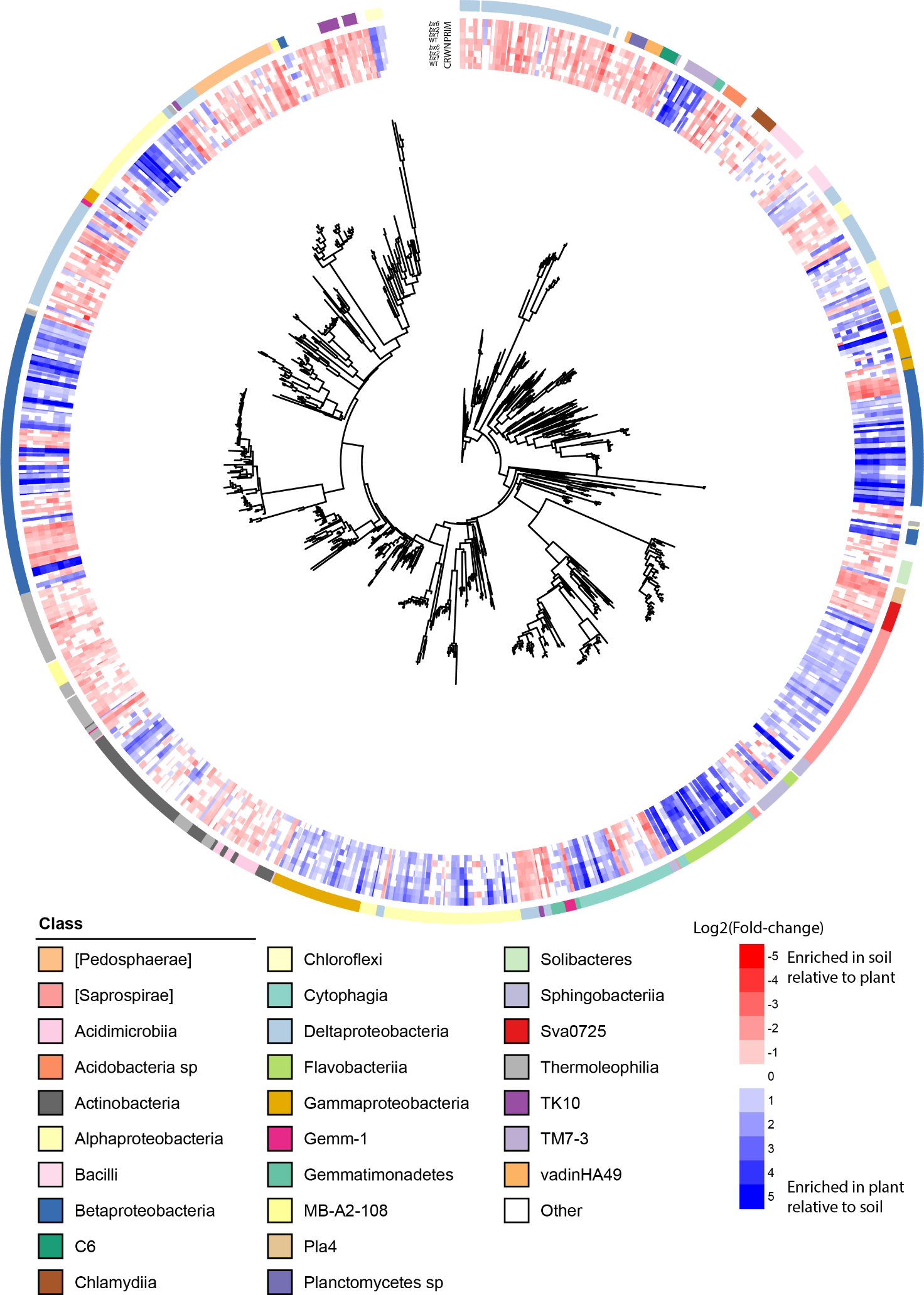
**Figure S10.** Bacterial OTUs that differ statistically in relative abundance between plant-free soil and root+rhizosphere samples. OTUs are arranged by phylogeny (class level). Shown are classes with a relative abundance greater than 1%. Heat map projections represent log2 fold-change values for each OTU. A complete list of statistically significant OTUs between all soil-genotype combinations is presented in Table S7.

**Figure S11.** Fungal OTUs that differ statistically in relative abundance between plant-free soil and root+rhizosphere samples. OTUs are arranged by taxonomy. Shown are classes with relative abundances greater than 1%. Heat map projections represent log2 fold-change values for each OTU. A complete list of statistically significant OTUs between all soil-genotype combinations is presented in Table S7.


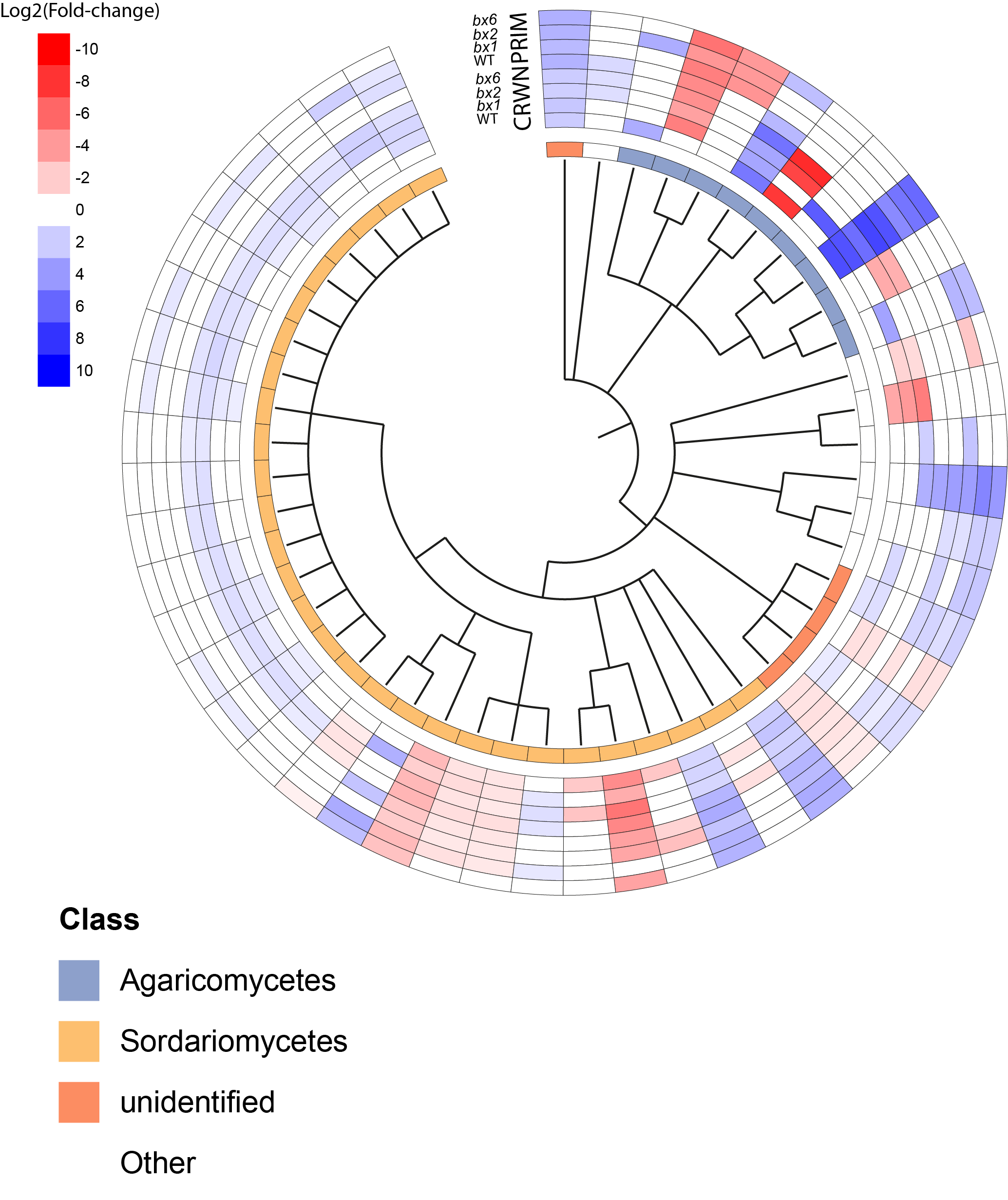


**Figure S12.** Volcano plots of bacterial (**a**) and fungal (**b**) OTUs. Plots show statistical significance (y-axis) against fold-change (x-axis) for all OTUs considered in the analysis. Red symbols indicate OTUs that show a statistically significant difference in relative abundance between *bx* mutant and WT roots (crown and primary; *p*-adjusted< 0.05, corrected for false discovery).


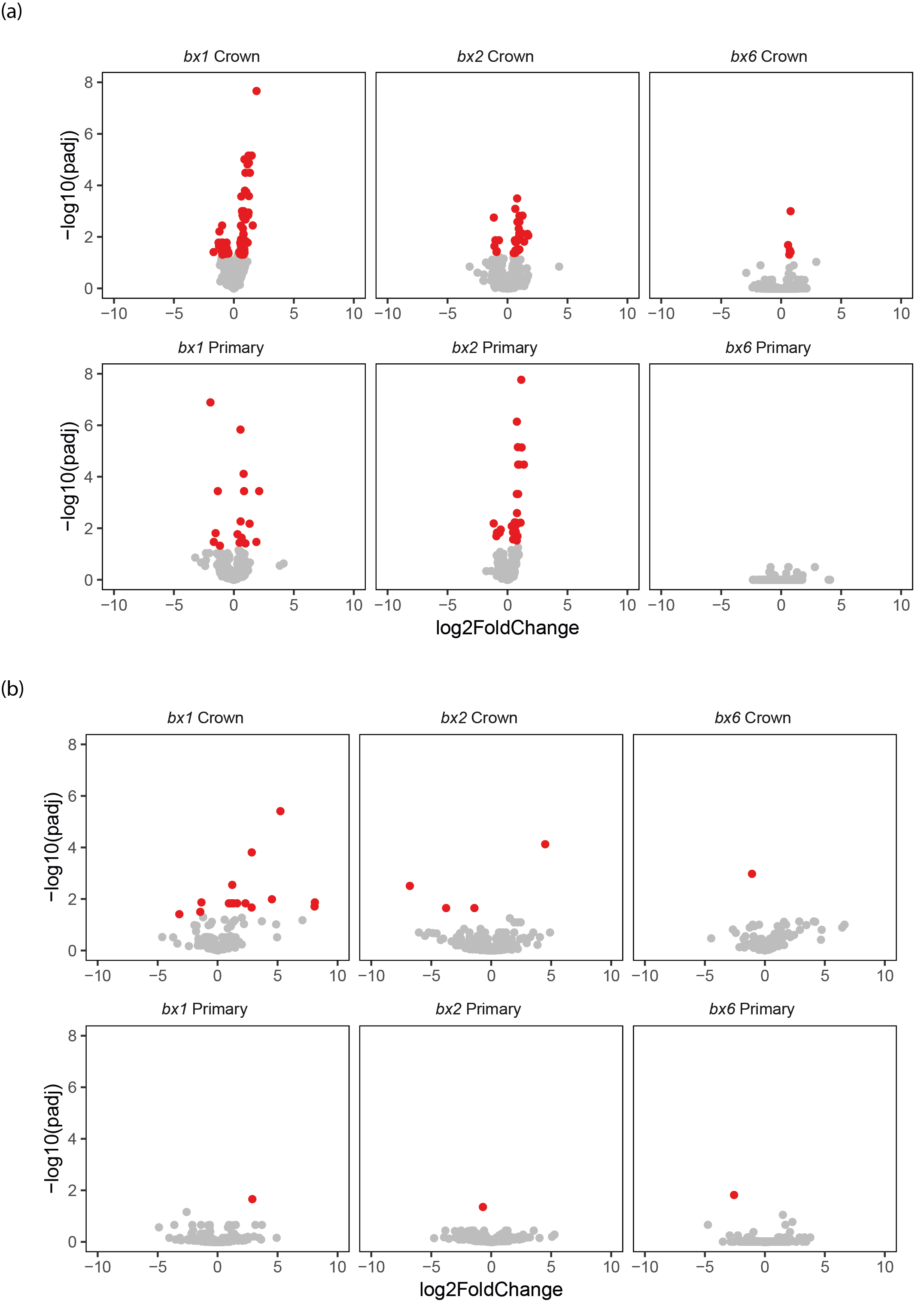


**Figure S13**. Correlation plots between LOG relative OTU abundance (x-axis) and LOG ion intensity (y-axis). The top 8 correlations for each cluster in Figure 4 are shown. Plot titles represent OTU ID and metabolite ID with Family and putative metabolic pathway. Symbols represent sample soil/root types (closed circles= primary roots, open triangles= crown roots). Colours represent genotypes (green = WT, red = *bx1*, purple = *bx2*, blue = *bx6*).


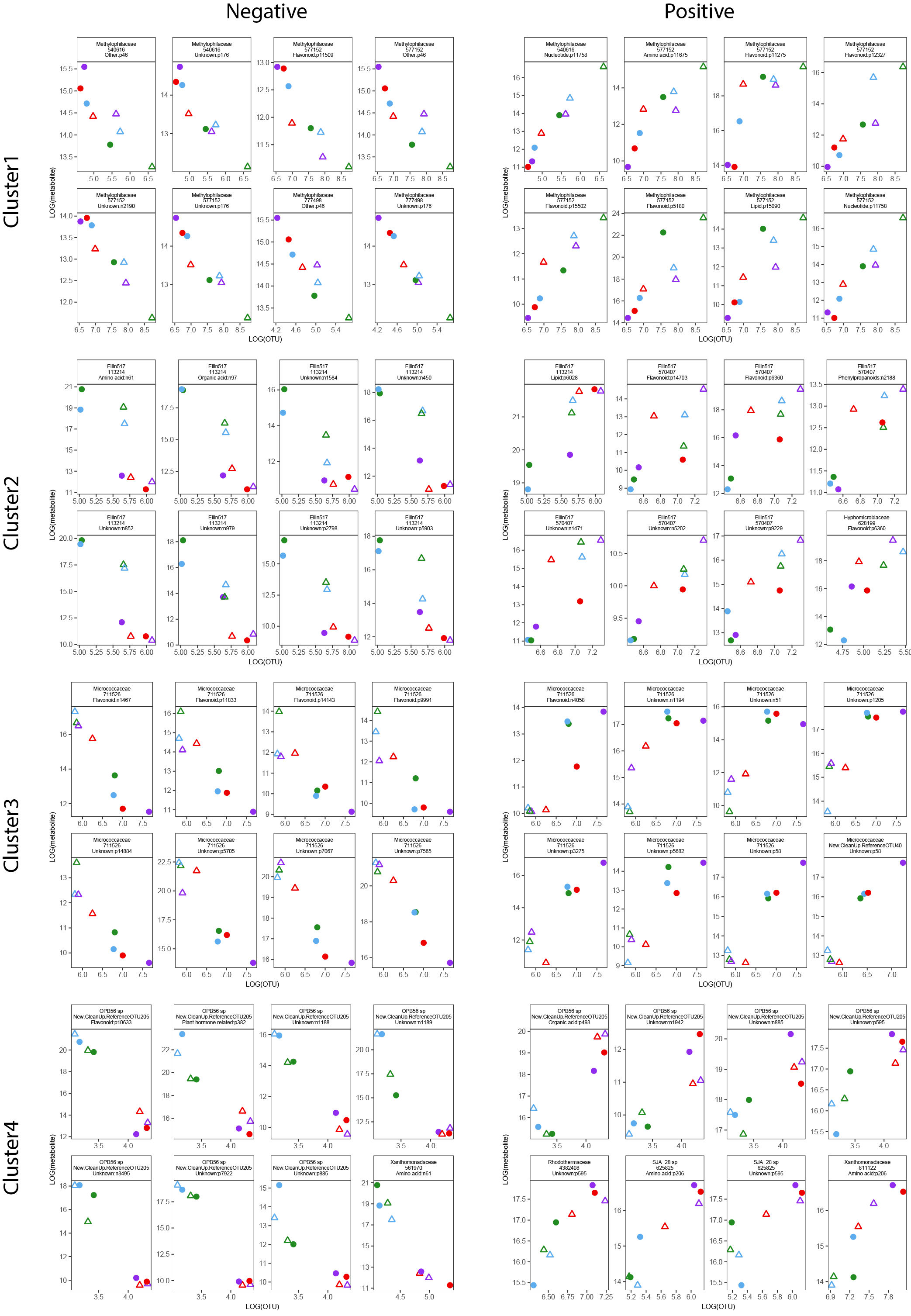

Supplement: Supplementary file 2 — Supplementary figures [file 41396_2019_375_MOESM2_ESM.docx]
