# Supplementary material for: Metabolic regulation of the maize rhizobiome by benzoxazinoids
Source: ISME J. 2019 Feb 22;13(7):1647–58. doi: 10.1038/s41396-019-0375-2 (PMC6592824; doi:10.1038/s41396-019-0375-2)
Supplement: Supplementary file 3 — Supplementary data file 1 [file 41396_2019_375_MOESM3_ESM.docx]

# Supplementary data file 1: re-analysis of data from this study and Hu *et al* (2018)

The details of the analysis protocols differed between the two studies, so the protocol of Hu *et al* (2018) was followed to enable direct comparisons to be drawn. However, both analyses produced similar results on the datasets. Data from Hu *et al* 2018 were downloaded and re-clustered with the data from this study (at 97% similarity). Taxonomies were assigned using RDP classifier. The clustering processes creates representative OTUs – these were named nOTU (new) to avoid confusion.

## Sequencing effort and community diversity.

The sequencing effort in terms of high quality reads is similar or greater for samples in this study (Cotton *et al*) compared with those of Hu *el al* (2018; Supplementary data file 1 – Fig. 1a). Measures of community diversity were also similar (Sup Fig 1b) although there was less variation in the Cotton *et al* samples.

| **a** |
| --- |
| **b** |
| **Fig. 1:** Sequencing effort (**a**) and alpha diversity (**b**). Samples with the suffix ‘sheffield’ are from Cotton *et al.* (present study), others are from Hu *et al* (2018). Results are means +/- SD of high quality sequences. |

## Multivariate statistical analysis.

Comparison of both datasets by principal coordinate analysis showed that the samples from this study (Cotton et al.) clustered together and were distinct from those of Hu *et al* (2018) (Supplementary data file 1 - Fig. 2).

| 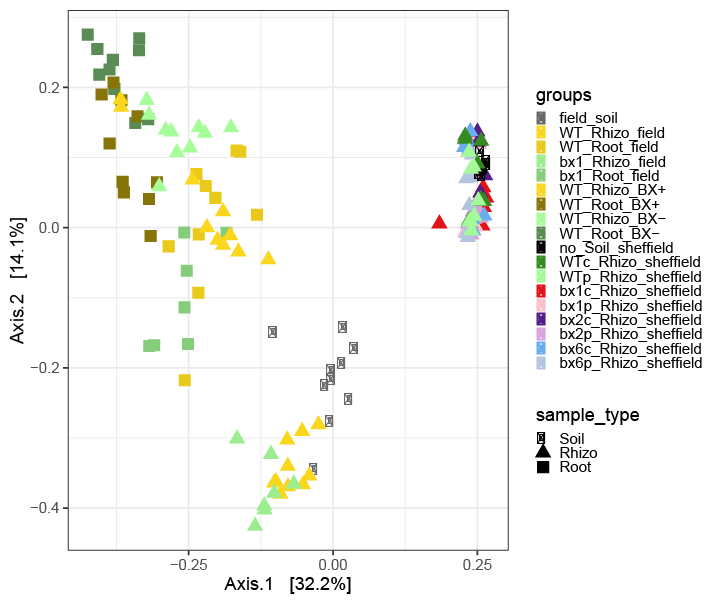 |
| --- |
| **Fig 2:** Principal coordinate analysis (PCoA) analysis of samples, using weighted Bray distances. |

***Taxonomic comparison.***

Comparisons of the operational taxonomic units (OTUs) in each group at the Class level shows broad similarities between the two studies, although the relative proportions differed (Supplementary data file 1 - Fig. 3).

| 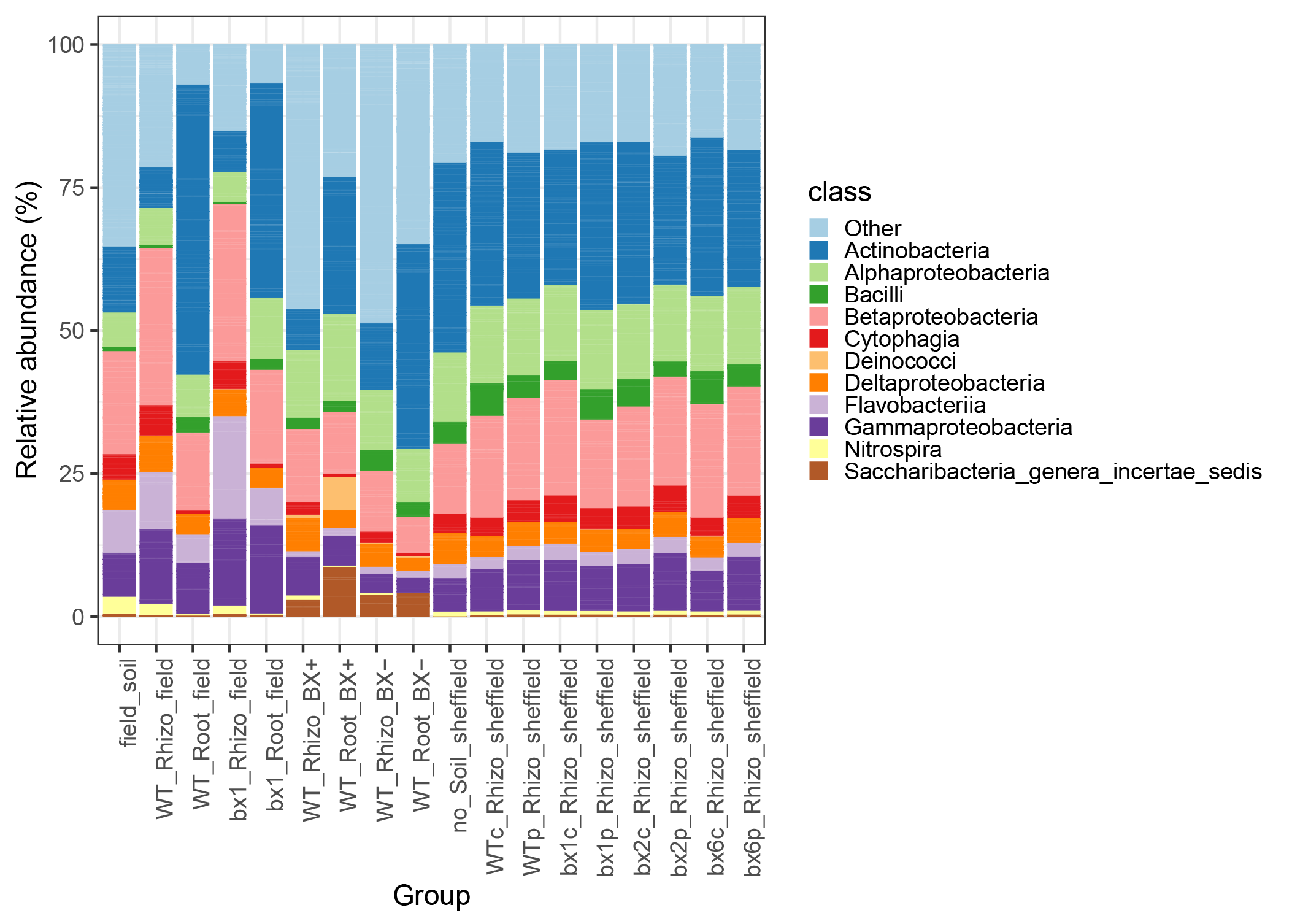 |
| --- |
| **Fig 3**: Relative abundance of OTUs at the Class taxonomic level. |

***Identification of shared OTUs***

To compare directly OTUs that were shared between studies, differentially abundant OTUs were identified using edgeR (analysis with DESEQ2 produced essentially the same results). Both data sets were re-analysed to allow common OTUs to be identified. In Hu *et al* (2018), only OTUs with a relative abundance >0.01% were reported. As the sequencing depth was greater in the current study (Cotton et al.), this was altered to >0.001%. Results are shown in Supplementary data file 1 – Tables 1a-1c. Differences were corrected for false discovery (FDR), using the Benjamini-Hochberg method.

**Table 1a**: re-analysis of Hu et al. (2018) field samples, showing OTU numbers different between WT and *bx1* plants (cv. B73).

| Rhizosphere samples | | |
| --- | --- | --- |
| Lower in WT | unchanged | Higher in WT |
| 4 | 177 | 8 |

| Root samples | | |
| --- | --- | --- |
| Lower in WT | unchanged | Higher in WT |
| 12 | 170 | 7 |

**Table 1b**: re-analysis of Hu et al. (2018) feedback samples, showing OTU numbers different between BX+ and BX- conditions.

| Rhizosphere samples | | |
| --- | --- | --- |
| Higher in BX+ | unchanged | Higher in BX- |
| 6 | 164 | 11 |

| Root samples | | |
| --- | --- | --- |
| Higher in BX+ | unchanged | Higher in BX- |
| 14 | 148 | 19 |

**Table 1c**: re-analysis of Cotton *et al.* (current study), showing OTU numbers different between WT and *bx1* plants (cv. W22).

|  | Higher in *bx1* | Unchanged | Higher in WT |
| --- | --- | --- | --- |
| Primary | 2 | 815 | 5 |
| Crown | 47 | 742 | 33 |

Overlap between the samples are shown by Venn diagrams in Supplementary data file 1 - Fig. 4 and Supplementary data file 1 - Table 2. For clarity, the comparisons with the field and feedback samples from Hu *et al.* (2018) are presented separately. As the OTUs from both studies were clustered together for the comparative analysis, multiple OTUs from the individual studies may match if the representative OTUs are not exactly the same. The closest match(es) in the individual studies are shown.

**Fig. 4**: Venn diagrams of OTUs showing significant differences between genotypes and treatments. Coloured sections indicate OTUs that are common between Hu *et al* (2018) and Cotton *et al* (present study). Left: comparison between the field data of Hu *et al.* (Field; Wt versus *bx1* mutant; cv. B73) and the controlled environment data of Cotton *et al.* (Sheffield; Wt versus *bx1* mutant; cv. W22). Right: comparison between the soil feedback data of Hu et al. (Feedback; BX+ versus BX-) and the controlled environment data of Cotton *et al.* (Sheffield; Wt versus *bx1* mutant; cv. W22).

**Table 2**: OTUs that are shared between Hu *et al* (2018) and Cotton *et al* (present study).

| OTU | Class | Family | Genus | Hu *et al* OTU | Cotton *et al* OTU |
| --- | --- | --- | --- | --- | --- |
| Field *vs* Sheffield | |  |  |  |  |
| nOTU47 | Betaproteobacteria | Methylophilaceae | Methylotenera | bOTU42 (100%) | 540616 (99%) |
| nOTU146 | Betaproteobacteria | Methylophilaceae | Methylotenera | bOTU84 (100%) | 777498 (100%) |
| nOTU315 | Gammaproteobacteria | Pseudomonadaceae | Cellvibrio | bOTU181 (100%) | 90390 (99%) |
| Feedback *vs* Sheffield | |  |  |  |  |
| nOTU339 | Alphaproteobacteria | Bradyrhizobiaceae | Tardiphaga | bOTU250/bOTU2097 (97%) | 567840/NCRefOTU33824 (99%) |
| nOTU305 | Betaproteobacteria | Oxalobacteraceae | Massilia | bOTU168 (100%) | 1074016/819708/548434/NCRefOTU30038 (99%) |
| nOTU11 | Betaproteobacteria | Oxalobacteraceae |  | bOTU63 (99%) | NCRefOTU983/2838672 (98%) |
| nOTU81 | Actinobacteria |  |  | bOTU225 (100%) | NCRefOTU48452 (99%) |
